# Two New Diterpenoids Formed by Transannular Diels–Alder Cycloaddition from the Soft Coral *Sarcophyton tortuosum*, and Their Antibacterial and PPAR-β Agonist Activities

**DOI:** 10.3390/md22120553

**Published:** 2024-12-10

**Authors:** Min Sun, Songwei Li, Jianang Zeng, Yuewei Guo, Changyun Wang, Mingzhi Su

**Affiliations:** 1 MOE Key Laboratory of Marine Drugs and Key Laboratory of Evolution and Marine Biodiversity, Institute of Evolution & Marine Biodiversity, School of Medicine and Pharmacy, Ocean University of China, Qingdao 266003, China; 21220813006@stu.ouc.edu.cn; 2 Shandong Laboratory of Yantai Drug Discovery, Bohai Rim Advanced Research Institute for Drug Discovery, Yantai 264117, China; zjianang05@163.com; 3 School of Medicine, Shanghai University, Shanghai 200444, China; songweili@shu.edu.cn; 4 Laboratory for Marine Drugs and Bioproducts, Qingdao Marine Science and Technology Center, Qingdao 266237, China

**Keywords:** soft coral, *Sarcophyton tortuosum*, polycyclic diterpenes, transannular Diels–Alder cycloaddition, antibacterial activity, PPAR-β agonist activity

## Abstract

Two new cembrane-derived tricyclic diterpenes belonging to the sarcophytin family, namely 4*a*-hydroxy-chatancin (**1**) and sarcotoroid (**2**), together with two known related ones (**3** and **4**), were isolated from the soft coral *Sarcophyton tortuosum* collected off Ximao Island in the South China Sea. The structures of the new compounds were elucidated by extensive spectroscopic analysis, a quantum mechanical nuclear magnetic resonance (QM-NMR) method, a time-dependent density functional theory electronic circular dichroism (TDDFT-ECD) calculation, X-ray diffraction analysis, and comparison with the reported data in the literature. A plausible biosynthetic pathway of compounds **1**–**4** was proposed, involving undergoing a transannular Diels–Alder cycloaddition. In the bioassay, the new compound **1** displayed significant inhibitory activities against the fish pathogens *Streptococcus parauberis* KSP28, oxytetracycline-resistant *Streptococcus parauberis* SPOF3K, and *Photobacterium damselae* FP2244, with MIC values of 9.1, 9.1, and 18.2 μg/mL, respectively. Furthermore, by conducting a luciferase reporter assay on rat liver Ac2F cells, compounds **1**, **3**, and **4** were evaluated for peroxisome proliferator-activated receptor (PPAR) transcriptional activity, and compound **3** showed selective PPAR-β agonist activity at a concentration of 10 μΜ.

## 1. Introduction

Soft corals of the genus *Sarcophyton* (phylum, Cnidaria; class, Anthozoa; subclass, Octocorallia; order, Alcyonaceae; family, Alcyoniidae) dominate many coral reef areas, which are characterized by a smooth mushroom-shaped appearance [1]. Chemical investigation studies have shown that the soft corals of this genus are furnished with a wealth of secondary metabolites, which possess complex/intriguing structural characteristics and exhibit a range of biological activities [2]. Notable metabolites include sesquiterpenes, diterpenes, diterpene dimers, prostaglandins, and steroids [3,4]. 

*Sarcophyton tortuosum* is widely distributed in the tropic oceans, especially the South China Sea, and has been continuously chemically investigated by our group for over a decade, leading to the isolation and characterization of a variety of structurally intriguing and biologically active diterpenes and diterpene dimers [5,6,7,8,9]. Among them, eunicellane-type diterpenoids, cembrane-type diterpenoids, and especially biscembranoids (diterpene dimers) are characteristic secondary metabolites of this soft coral. Cembranoids, diterpenes with a 14-membered macrocycle, have been known to be common intermediates in the biogenesis of polycyclic diterpenes and biscembranoids by intramolecular 2 + 2 cycloaddition and intermolecular 4 + 2 (Diels–Alder) cycloaddition [10,11,12], respectively. However, there are no examples of the natural product polycyclic diterpenoid formed by intramolecular 4 + 2 (Diels–Alder) cycloaddition involving cembranoids, and only a few examples of biomimetic syntheses of natural products with a Diels–Alder reaction [13,14], which piqued our great interest in searching for more evidence of natural products produced by intramolecular Diels–Alder cycloaddition involving soft corals.

**Figure 1:**
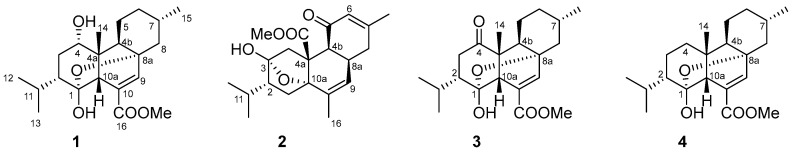
Chemical structures of compounds **1**–**4**.

In this work, a chemical investigation of a new collection of soft coral *S. tortuosum* from Ximao Island, Hainan Province, China, resulted in the isolation and full characterization of two previously undescribed compounds, **1** and **2**, along with two known compounds, **3** and **4** (Figure 1). Herein, the isolation, structure elucidation, plausible biosynthetic pathway, and biological evaluation of these isolated compounds will be described.

## 2. Results and Discussion

Freshly collected specimens of *S. tortuosum* were frozen to –20 °C, before being exhaustively extracted with acetone. The Et_2_O-soluble portion of the acetone extract of the title animal was subjected to repeated chromatography, including with silica gel, Sephadex LH-20, and RP-HPLC, to yield four compounds (**1**–**4**). The known compounds **3** and **4** were readily identified as 4-oxochatancin (**3**) [15] and (+)-chatancin (**4**) [16], respectively, by direct comparison of their NMR spectroscopic data and specific rotation value with those reported in the literature.

Compound **1** was isolated as a colorless crystal. Its molecular formula of C_21_H_32_O_5_ was established by the quasi-molecular ion peak at *m/z* 387.2129 ([M+Na]^+^, calc. 387.2147 for C_21_H_32_O_5_Na) in the HR-ESIMS spectrum, indicating six degrees of unsaturation. The IR spectrum displayed strong absorption at 3460 cm^−1^ and 1712 cm^−1^, suggesting the presence of hydroxyl and carbonyl groups. The ^1^H NMR, ^13^C NMR, and DEPT spectra of **1** suggested the presence of one trisubstituted double bond [*δ*_H_ 7.27 (1H, s, H-9), *δ*_C_ 145.11 (CH, C-9), and *δ*_C_ 134.82 (qC, C-10)], one carbonyl carbon atom [*δ*_C_ 165.41 (qC, C-16)], and one oxymethine [*δ*_H_ 3.53 (1H, m, H-4), *δ*_C_ 77.0 (CH, C-4)]. Consequently, the remaining four degrees of unsaturation led to the discovery that compound **1** was a tetracyclic structure.

A detailed comparison revealed that the NMR data (Table 1) of compound **1** were almost identical to those of the co-occurring compounds **3** and **4**, which had been previously isolated from the tropical marine slug *Phyllodesmium longicirrum* that preys on soft corals of the genus *Sarcophyton*. The only difference between **1** and **3** was that the carbonyl group at the C-4 position in **3** was replaced by a hydroxyl group in **1**, which was evidenced by the 2 mass unit difference in their ESIMS data. Thus, the planar structure of **1** was determined as shown in Figure 1.

**Figure 2:**
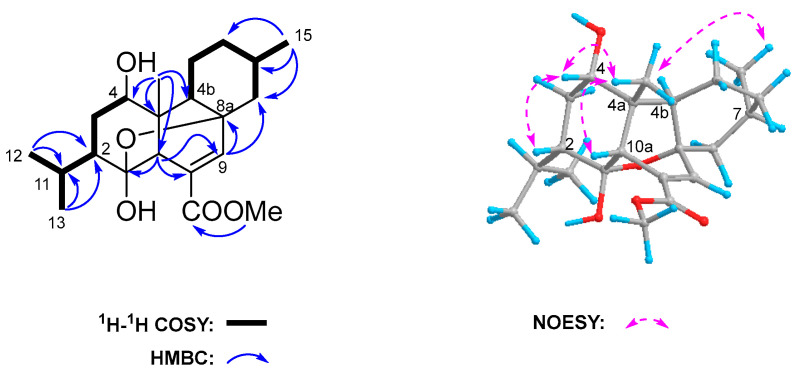
^1^H–^1^H COSY, selected key HMBC, and NOESY correlations of **1**.

In the NOESY spectrum, there was a clear correlation between H-4 (*δ*_H_ 3.53, m), CH_3_-14 (*δ*_H_ 0.85, s), H-2 (*δ*_H_ 1.56), and H-10a (*δ*_H_ 2.79, d, *J* = 1.73), indicating that H-2, H-4, CH_3_-14, and H-10a are on the same side of the molecule, and randomly assigned as having a *b*-orientation (Figure 2). Subsequently, the interactions of H-4b (*δ*_H_ 1.60) and CH_3_-15(*δ*_H_ 1.00, d, *J* = 6.3) revealed that these protons and/or proton-bearing groups were positioned at the other side of the molecule, thus having an *a*-orientation. In the light of this observation, the relative configurations of C-2, C-4, C-4a, C-4b, C-7, and C-10a in **1** were assigned to be 2S*, 4S*, 4aR*, 4bR*, 7*S*, and 10aR*, respectively. In addition, the relative stereochemistry of C-1 and C-8a in **1** was tentatively determined to be the same 1R* and 8aR* as that of **3**, on basis of their highly similar ^13^C NMR data.

**Figure 3:**
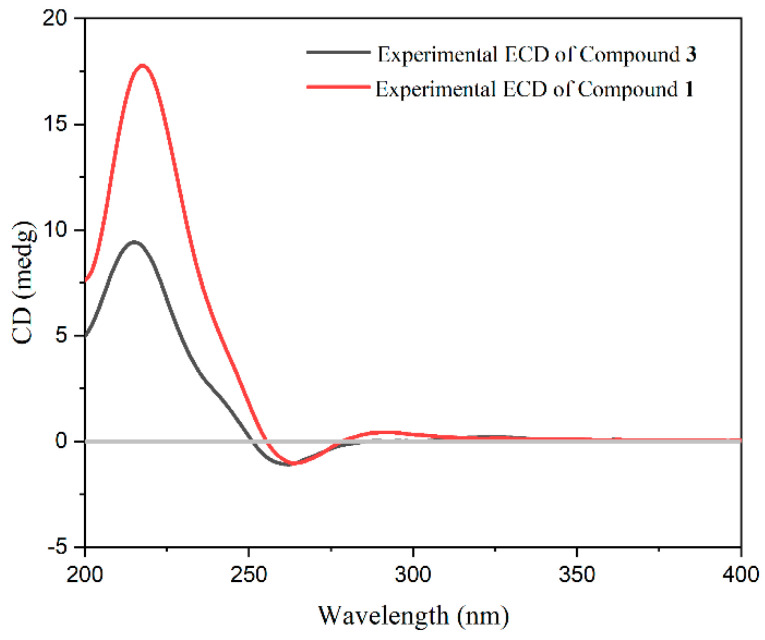
Experimental ECD spectra of **1** (red) and **3** (black).

The absolute configuration of **1** was determined by comparing its ECD spectrum with that of **3**. As shown in Figure 3, the cotton effects in the ECD spectra of the two compounds are consistent with each other. Therefore, the absolute configuration of **1** was ultimately determined as 1*R*, 2*S*, 4*S*, 4a*R*, 4b*R*, 7*S*, 8a*R*, 10a*R*.

**Figure 4:**
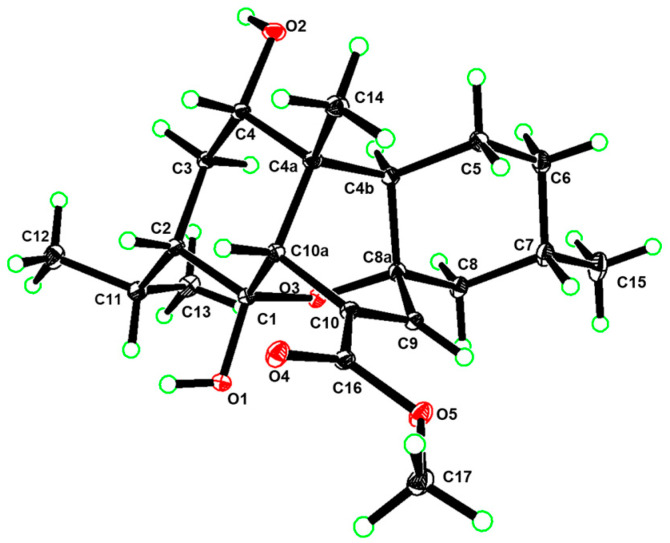
ORTEP drawing of **1** (the displacement ellipsoids are drawn at the 50% probability level).

In order to unambiguously confirm the structure and absolute configuration of **1**, suitable single crystals of **1** in MeOH were obtained. X-ray crystallographic analysis using Cu K*α* radiation (λ = 1.54178 Å) firmly validated the planar structure of **1** and determined its absolute configuration as 1*R*, 2*S*, 4*S*, 4a*R*, 4b*R*, 7*S*, 8a*R*, 10a*R*, with a Flack parameter of 0.08(7) (Figure 4, CCDC 2384793). Consequently, the structure of **1** was elucidated as depicted in Figure 1, and named as 4*a*-hydroxy-chatancin.

Sarcotoroid (**2**) was isolated as a colorless oil, which possessed the molecular formula of C_21_H_28_O_5_, as assigned by the HR-ESIMS protonated molecular ion peak at *m/z* 361.2004 ([M+H]^+^, calc. for C_21_H_29_O_5_, 361.2010), requiring eight degrees of unsaturation. The IR spectrum of **2** showed absorption bonds at 3300 cm^–1^ and 1727 cm^–1^, suggesting the presence of hydroxy and ester carbonyl functionalities. The 1D and 2D NMR spectra of **2** revealed the presence of two trisubstituted double bonds [*δ*_H_ 5.86 (1H, s, H-6), *δ*_C_ 161.51 (qC, C-7), and *δ*_C_ 127.46 (CH, C-6); *δ*_H_ 5.89 (1H, s, H-9), *δ*_C_ 134.19 (qC, C-10), and *δ*_C_ 135.25 (CH, C-9)], one carbonyl group [*δ*_C_ 197.91 (qC, C-5)], one methyl ester group [*δ*_H_ 3.71 (3H, s, H-17) and *δ*_C_ 175.33 (qC, C-14)], and one hemiketal group [*δ*_C_ 106.36 (qC, C-3)]. The above functionalities accounted for four degrees of unsaturation, suggesting that compound **2** must possess a tetracyclic ring system.

**Figure 5:**
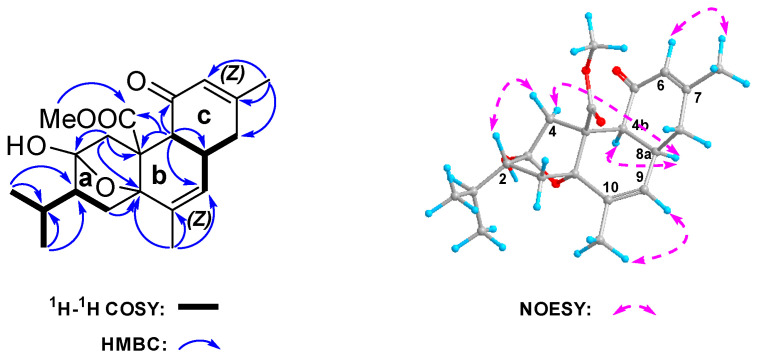
^1^H–^1^H COSY, selected key HMBC and NOESY correlations of **2**.

Detailed analysis of ^1^H–^1^H COSY spectrum of **2** revealed two sequential fragments (Figure 5) by the clear correlations of H-4b [*δ*_H_ 2.91 (d, *J* = 13.0)]/H-8a [*δ*_H_ 3.02, m]/H-8 [*δ*_H_ 2.25, m]/H-9 [*δ*_H_ 5.89, m], and CH_3_-12 [*δ*_H_ 0.88 (d, *J* = 6.7)]/CH_3_-13[*δ*_H_ 0.90 (d, *J* = 6.7)]/H-11 [*δ*_H_ 1.98, m]/H-2 [*δ*_H_ 2.18, m]/H_2_-1 [*δ*_H_ 1.82 (dd, *J* = 13.1, 6.5), *δ*_H_ 1.46 (dd, *J* = 13.1, 8.3)]. The key HMBC correlations from H-4 to C-3, C-4a, and C-10a, and from H-1 to C-2, C-3, C-10a, and C-4a, indicated the presence of ring (**a**) in the structure. The second ring (**b**) was confirmed by HMBC correlations from H-4b to C-4a, C-8a, and C-9, as well as from CH_3_-16 to C-9, C-10, and C-10a. The diagnostic HMBC correlation from H-4b to C-8a, C-8, and C-5, and from CH_3_ to C-6, C-7, and C-8, agreed with the presence of the third ring (**c**). Considering the downfield shift of the two quaternary carbons C-3 (*δ*_C_ 106.36) and C-10a (*δ*_C_ 81.47), the remaining one degree of unsaturation must be given by the formation of an oxygen bridge between C-3 and C-10a. Finally, the planar structure of **2** was determined as shown in Figure 5.

**Figure 6:**
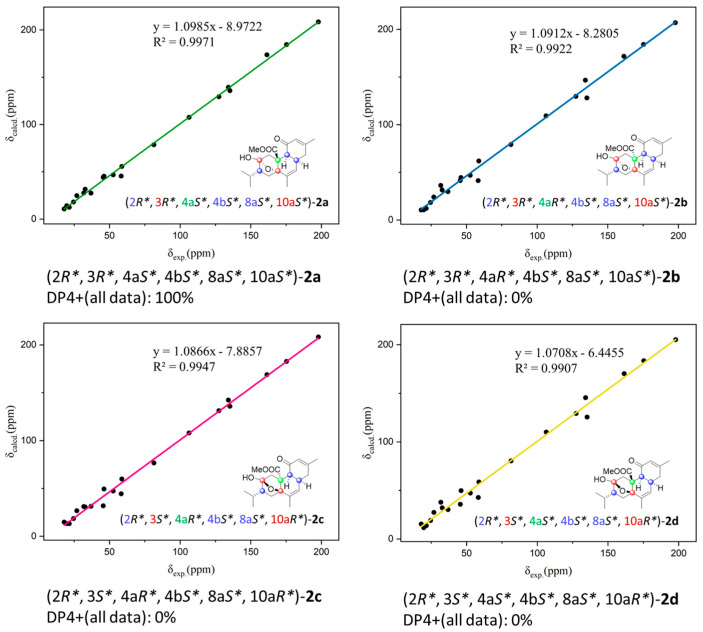
Regression analysis of experimental vs. calculated ^13^C NMR chemical shifts of **2a**–**2d** using DP4+ method.

**Figure 7:**
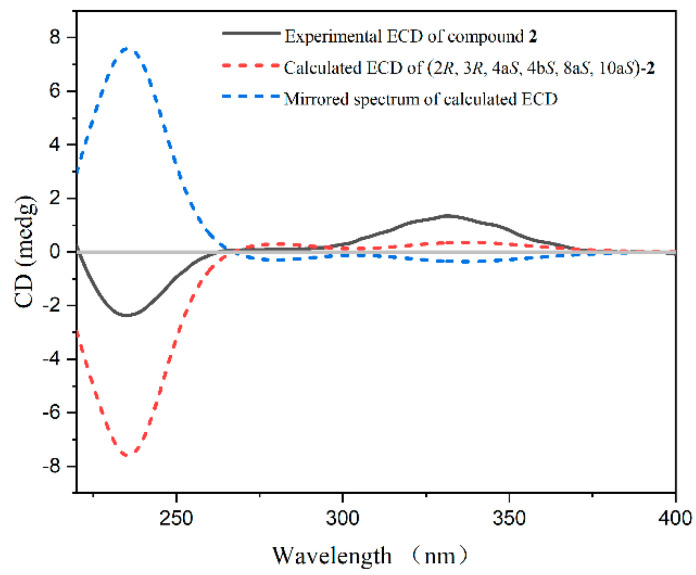
Experimental spectrum of **2** (black solid line), and calculated ECD spectra of (2*R*, 3*R*, 4a*S*, 4b*S*, 8a*S*, 10a*S*)-**2** (red dashed line) and (2*S*, 3*S*, 4a*R*, 4b*R*, 8a*R*, 10a*R*)-**2** (blue dashed line).

The observed NOESY correlations between H-6 (*δ*_H_ 5.86)/CH_3_-15 (*δ*_H_ 1.96) and H-9 (*δ*_H_ 5.89)/CH_3_-16 (*δ*_H_ 1.86) demonstrated the *Z* geometries of the double bonds at Δ^6,7^ and Δ^9,10^. The NOE correlations of H-4b/H-8a suggested that H-4b and H-8a were in the same orientation. The NOE correlations of H-8a/H*a*-4 and H*b*-4/H-2 indicated that H-2 and H-8a were in an opposite orientation. Thus, the relative configurations of C-4b, C-8a, and C-2 were assigned as 2R*, 4bS*, and 8aS*. Due to the advantageous conformation of the five-membered ring in the envelope form, the relative configuration of C-3, C-10a was determined to be 3R* and 10aS*. However, the relative configuration of C-4a remained a challenging task, due to the absence of related NOESY correlations. Therefore, the quantum mechanical–nuclear magnetic resonance (QM-NMR) method, which has been widely used to define the relative configuration of multi-stereogenic centers of natural products [17], was applied. Four possible candidate structures, namely **2a** (2R*, 3R*, 4aS*, 4bS*, 8aS*, 10aS*), **2b** (2R*, 3R*, 4aR*, 4bS*, 8aS*, 10aS*), **2c** (2R*, 3S*, 4aR*, 4bS*, 8aS*, 10aR*), and **2d** (2R*, 3S*, 4aS*, 4bS*, 8aS*, 10aR*), were calculated for their theoretical NMR data. Firstly, conformational searches on the four possible candidate diasteroisomers were performed. Then, geometrical optimization at the DFT level was undertaken using the B3LYP functional with the 6-31G* basis set. Next, NMR calculations were carried out at the PCM/mPW1PW91/6-311+G** level, as recommended for DP4+ [18]. NMR shielding constants were calculated using the GIAO approach [19,20,21,22]. Finally, the shielding constants were averaged over the Boltzmann distribution obtained for each stereoisomer, and correlated with the experimental data. The experimental NMR data of **2** gave the best match to that of **2a**, with over 99% probability (see the Supporting Information for details). Each isomer was statistically analyzed by linear regression of the calculated shifts (*δ*_calcd_) against the experimental ones (*δ*_expt_). In Figure 6, we report the statistical parameters of four possible candidate structures, where structure **2a** has an R^2^ (0.9971) coefficient close to **1**. Therefore, the relative configuration of compound **2** was ultimately determined as (2R*, 3R*, 4aS*, 4bS*, 8aS*, 10aS*). Moreover, the TDDFT-ECD method was also performed to determine the absolute configuration of **2** [23,24]. As shown in Figure 7, the calculated ECD curve of (2*R*, 3*R*, 4a*S*, 4b*S*, 8a*S*, 10a*S*)-**2** fits in good agreement with the experimental ECD curve of **2**. Finally, the absolute configuration of **2** was assigned as 2*R*, 3*R*, 4a*S*, 4b*S*, 8a*S*, 10a*S*, as shown in Figure 1.

**Scheme 1:**
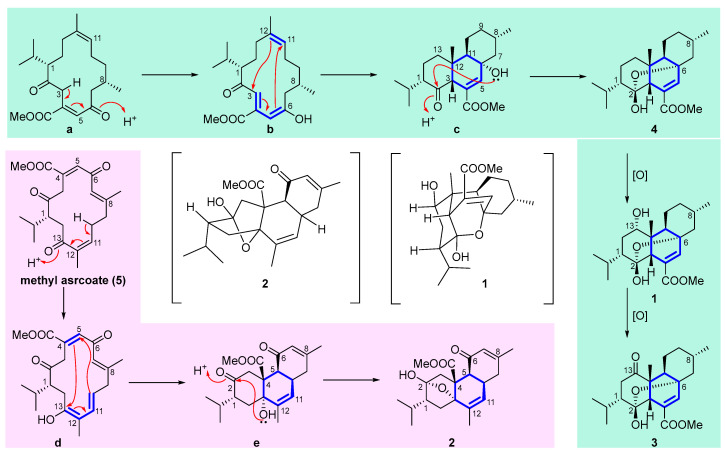
Plausible biosynthetic pathways of **1**–**4** (cembranoid numbering throughout pathways).

It is worth pointing out that the structural similarities between compounds **1**–**4**, and their co-occurrence in the same sample, suggested that they may originate from the same biogenetic pathway. A plausible biosynthetic pathway for structural correlations among the related diterpenoids **1**–**4** was proposed. As shown in Scheme 1, compounds **1**, **3**, and **4** herein probably have the same precursor macrocyclic diketone **a**. Firstly, the C-6 of **a** undergoes enol interconversion, resulting in the formation of conjugated double bonds at Δ^3,4^ and Δ^5,6^ in **b**. Subsequently, these conjugated double bonds participate in a transannular Diels–Alder cycloaddition with the double bond at Δ^11,12^, leading to the formation of a core structure featuring an uncommon 6/6/6-tricyclic ring system. Finally, the lone pair electrons of the oxygen atom in 6-OH attack the C-2 carbon atom, resulting in the formation of **4** with an epoxy bridge between C-6 and C-2. Following two steps of oxidation, compounds **1** and **3** are ultimately obtained. Similarity, the precursor methyl sarcoate (**5**) [25] undergoes enol interconversion to yield **d**, and the conjugated double bonds at Δ^10,11^ and Δ^12,13^ of **d** undergo a transannular Diels–Alder cycloaddition with the double bonds at Δ^4,5^ to generate **e**, followed by the nucleophilic attack of hydroxyl group 13-OH on C-2, forming **2**.

In the bioassay, all the isolated compounds were tested for their antibacterial effects. *Streptococcus parauberis* is the primary pathogen responsible for fish-borne streptococcal disease, which is widely distributed and highly pathogenic, causing significant economic losses in fish aquaculture worldwide. *S. parauberis* can infect a diverse range of fish, including both freshwater and farmed or wild species, causing them to exhibit clinical symptoms similar to septicemia. However, these strains have gradually developed drug resistance, including multidrug resistance. The *S. parauberis* SPOF3K strain is a drug-resistant isolate from aquaculture that exhibits significant resistance to tetracycline and oxytetracycline, with MICs of over 24 μg/mL and 12.42 μg/mL, respectively. Notably, compound **1** exhibited good antibacterial activity against the *S. parauberis* KSP28 and oxytetracycline-resistant *S. parauberis* SPOF3K, with an MIC value of 9.10 μg/mL, which is superior to the antibacterial effect of oxytetracycline (MIC = 12.42 μg/mL) (Table 2).

*Photobacterium damselae* (formerly *Vibrio damsela*) is a halophilic bacterium associated with marine environments, known to cause skin ulcers in damselfish. It is a primary pathogen responsible for ulcers and hemorrhagic septicemia in various marine species, including dolphins, sharks, and shrimp, as well as both wild and cultivated fish. Additionally, this pathogen can lead to fatal infections in humans [26]. Compound **1** showed significant antibacterial activities against the *P. damselae* FP2244, with an MIC value of 18.21 μg/mL.

Furthermore, the isolated compounds were tested for antibacterial activity against *Pseudomonas aeruginosa*, *Staphylococcus aureus*, *Escherichia coli*, *Enterobacter cloacae*, *Enterobacter hormaechei*, *Aeromonas sabnonicida*, *Photobacterium halotolerans*, *Lactococcus garvieae* FP MP5245, and several strains of vancomycin-resistant *Enterococcus faecium* (G1, G4, G7, and G13), as well as *Streptococcus agalactiae*, *Edwardsiella piscicida* TH1, *Vibrio parahaemolyticus*, and *Vibrio alginolyticus*. However, these results were negative at a concentration of 100 μM.

**Figure 8:**
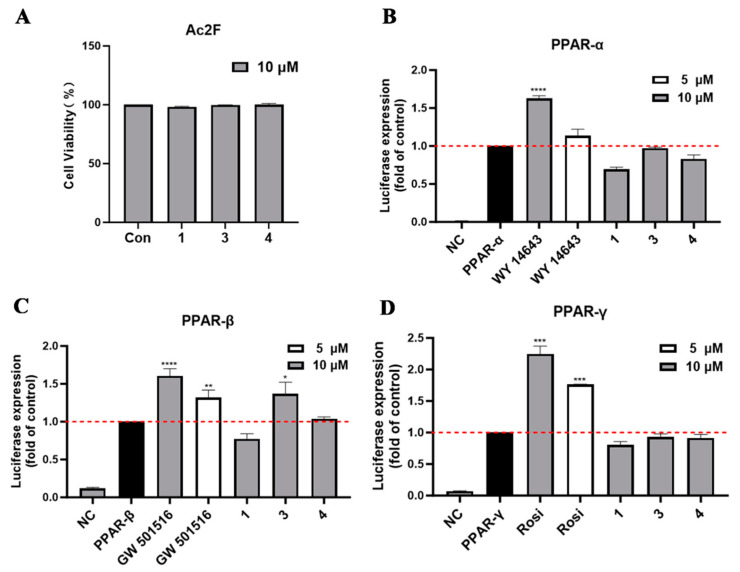
In vitro PPAR activation by the isolated compounds **1**, **3**, and **4** in rat liver Ac2F cell line. (**A**) Cytotoxicity of compounds **1**, **3**, and **4**, on rat liver Ac2F cells. (**B**) In vitro assay of PPAR-α activation by compounds **1**, **3**, and **4.** (**C**) In vitro assay of PPAR-β activation by compounds **1**, **3**, and **4.** (**D**) In vitro assay of PPAR-γ activation by compounds **1**, **3**, and **4.** NC: blank control, transfected with a plasmid containing PPRE and pcDNA; PPARα/β/γ: negative control, transfected with a plasmid containing PPRE and pFlag-PPAR-α, β, or γ, respectively. WY14643, GW501516, and Rosi (rosiglitazone) were used as positive controls for PPAR-α, PPAR-β, and PPAR-γ, respectively, to monitor the activation of the luciferase reporter. Luciferase expressions (folds of the control) are presented as mean ± SD (n ¼ 3). * *p* < 0.05, ** *p* < 0.01, *** *p* < 0.005, and **** *p* < 0.001.

Peroxisome proliferator-activated receptors (PPAR) include PPAR-α, PPAR-β, and PPAR-γ isotypes, which are transcription factors that regulate gene expression upon ligand activation. PPAR-α enhances cellular fatty acid uptake, esterification, and trafficking, while also regulating genes involved in lipoprotein metabolism. PPAR-β stimulates lipid and glucose utilization by increasing mitochondrial function and fatty acid desaturation pathways. Meanwhile, PPAR-γ promotes fatty acid uptake, triglyceride formation, and storage in lipid droplets, which enhances insulin sensitivity and glucose metabolism [27]. The compounds **1**, **3**, and **4** were evaluated for their PPAR transcriptional activity using a luciferase assay (Figure 8). As shown in Figure 8A, these compounds were first evaluated for their cytotoxicity against Ac2F cells. Compounds **1**, **3**, and **4** demonstrated no cytotoxic effects on the Ac2F cells at a concentration of 10 μM after 24 h of treatment. Notably, compound **3** exhibited significant and selective PPAR-β agonist activity at a concentration of 10 μM, comparable to that of the positive control GW501516 at 5 μM.

## 3. Materials and Methods

### 3.1. General Experimental Procedures

Melting points were measured with an X-4 digital micro-melting-point apparatus. The X-ray measurement was made using a Bruker D8 Venture X-ray diffractometer with Cu Kα radiation (Bruker Biospin AG, Fällanden, Germany). Optical rotations were measured with a Perkinelmer 241 MC polarimeter. The IR spectrum was recorded with a Nicolet iS50 spectrometer (Thermo Fisher Scientific, Madison, WI, USA). ^1^H and ^13^C NMR spectra were acquired using a Bruker DRX-600 spectrometer (Bruker Biospin AG, Fällanden, Germany). The chemical shifts are reported with the residual CDCl_3_ (*δ*_H_ 7.26 ppm) as the internal standard for ^1^H-NMR spectrometry, and CDCl_3_ (*δ*_C_ 77.26 ppm) for ^13^C-NMR spectrometry. The HR-ESI-MS spectra were recorded with a ZenoTOF7600 mass spectrometer (SCIEX). Commercial silica gel (Qingdao Haiyang Chemical Co., Ltd., Qingdao, China; 200–300 and 300–400 mesh) and Sephadex LH-20 gel (Amersham Biosciences, Sweden) were used for column chromatography (CC), and precoated silica gel plates (G60 F-254, Yan Tai Zi Fu Chemical Group Co., Yantai, China) were used for analytical TLC. Spots were detected on the TLC under UV light, or by heating after spraying with anisaldehyde H_2_SO_4_ reagent. Reversed phase (RP) HPLC was performed on an Agilent 1260 series liquid chromatograph, equipped with a DAD G1315D detector, at 210 nm (Agilent, Santa Clara, CA, USA). An Agilent semi-preparative XDB-C18 column (5 μm, 250 × 9.4 mm) was employed for the purification. All the solvents used for the CC and HPLC were of analytical grade (Shanghai Chemical Reagents Co., Ltd., Shanghai, China) and chromatographic grade (Dikma Technologies Inc., Beijing, China), respectively.

### 3.2. Animal Materials

The soft coral *S. tortuosum* was collected from Ximao Island, Hainan Province, China, in 2023, at a depth of –20 m, and identified by Professor Xiu-Bao Li from Hainan University. A voucher specimen (No. 23-YT-20) is available for inspection at the Shandong Laboratory of Yantai Drug Discovery.

### 3.3. Extraction and Isolation

The frozen animals (800.4 g, dry weight after extraction) were cut into pieces and extracted exhaustively with acetone at room temperature (4 × 2L). The organic extract was evaporated to produce a brown residue, which was then partitioned between diethyl ether (Et_2_O) and H_2_O. The Et_2_O solution was concentrated under reduced pressure to obtain a dark brown residue (58.0 g), which was classified by silica gel column chromatography (200–300 mesh) and eluted with a step gradient [0–100% Et_2_O in petroleum ether (PE) solution] to obtain six fractions (A–G). Fraction D (2.1 g) was subjected to chromatography on a Sephadex LH-20 column using a petroleum ether (PE)/CH_2_Cl_2_/MeOH (2:1:1) elution system, yielding subfractions DA to DC. Purification of the DB subfraction was performed using Sephadex LH-20 (CH_2_Cl_2_), followed by silica gel column chromatography with a PE/Et_2_O (20:1) elution, yielding four subfractions (DBAA–DBAD). Further purification of DBAB by RP-HPLC [CH_3_CN/H_2_O (85:25), 3.0 mL/min] led to the isolation of compound **4** (4 mg, t_R_ = 29 min). Fraction F (2.3 g) was purified by chromatography on the Sephadex LH-20, as described above, yielding subfractions FBA–FBE and FCA–FCH. Subfraction FBA was subjected to silica gel column chromatography, eluting with PE/Et_2_O (15:1), to give five subfractions (FBAA–FBAE). Further purification of subfraction FBAB by semi-preparative RP-HPLC (CH_3_CN/H_2_O, 70:30) yielded compound **3** (2 mg, t_R_ = 23.5 min). Further purification of subfraction FCD by semi-preparative RP-HPLC (CH_3_CN/H_2_O, 52:48) yielded compound **1** (1.2 mg, t_R_ = 11 min), and purification of subfraction FCF by semi-preparative RP-HPLC (CH_3_CN/H_2_O, 45:55) yielded compound **2** (1.7 mg, t_R_ = 16 min).

### 3.4. Spectroscopic Data of Compounds

4*a*-hydroxy-chatancin (**1**): colorless crystal, (m. p. 122–124 °C), ${\left[\alpha \right]}_{D}^{20}$–2 (c 0.10, MeOH); IR (KBr): ν_max_ 3460, 2952, 2925, 2868, 1712, 1268 cm^−1^; for ^1^H and ^13^C NMR spectroscopic data, see Table 1; HR-ESI-MS *m/z* 387.2129 ([M+Na]^+^; calculated for C_21_H_32_O_5_Na, 387.2147).

Sarcotoroid (**2**): colorless oil, ${\left[\alpha \right]}_{D}^{20}$–16 (c 0.10, MeOH); IR (KBr): ν_max_ 3300, 2952, 2918, 2850, 1727, 1627 cm^−1^; for ^1^H and ^13^C NMR spectroscopic data, see Table 1; HR-ESI-MS *m/z* 361.2004 ([M+H]^+^; calculated for C_21_H_29_O_5_, 361.2010).

### 3.5. Calculation Section

For the QM-NMR calculations of compounds in Macro model 9.9.223 software [Schrodinger, http://www.schrodinger.com/MacroModel (accessed on 11 November 2024)], a conformational search was performed by using the torsional sampling (MCMM) approach and the OPLS_2005 force field, applying an energy window of 21 kJ/mol (5.02 kcal/mol). We reoptimized the conformers of populations exceeding 1% Boltzman population at the B3LYP/6-31G (d, p) level. Subsequently, nuclear magnetic resonance calculations were performed at the PCM/mPW1PW91/6-311+G (d, p) level using Gaussian 09 [28]. The shielding constant of NMR was calculated using the GIAO method [19,20,21,22]. Finally, we calculated the average shielding constant for each stereoisomer Boltzmann distribution, and correlated it with the experimental data.

Regarding the TDDFT-ECD calculations of compounds, a conformational search was carried out according to the general protocols previously described for the QM-NMR calculation. We reoptimized the conformers above 1% Boltzmann population, and used the IEFPCM solvent model for the TDDFT-ECD calculations, using Gaussian 09 at the theoretical B3LYP/6-311G (d, p) level. Finally, the SpecDis 171 software was used to obtain the calculated ECD spectrum and visualize the results [29].

### 3.6. X-ray Crystallographic Analysis for Compound ***1***

The crystallographic data were collected with a Bruker D8 Venture diffractometer equipped with Cu Kα radiation (λ = 1.54178 Å). The structures were solved with the ShelXT structure solution program, using Intrinsic Phasing, and refined with the ShelXL refinement package, using Least Squares minimization.

Compound **1**: colorless crystals (m.p. 122–124 °C), monoclinic, C_21_H_34_O_6_, Mr = 382.48, (including a water molecule) crystal size 0.2 × 0.15 × 0.04 mm^3^, space group C2, a = 19.2786(5) Å, b = 11.3201(3)Å, c = 10.4695(3) Å, V = 2061.47(10) Å^3^, Z = 4, ρcalc = 1.232 g/cm^3^, F (000) = 832.0. Independent reflections: 3908 [R_int_ = 0.0656, R_sigma_ = 0.0341]. R_1_ = 0.0331, wR_2_ = 0.0810 reflections with I ≥ 2σ (I), R_1_ = 0.0331, wR_2_ = 0.0810 for all unique data. Flack parameter: 0.08(7). The crystals of **1** were recrystallized from MeOH. The above crystal data were deposited in the Cambridge Crystallographic Data Centre (CCDC) and assigned the accession number (CCDC 2384793).

### 3.7. Antibacterial Activity Bioassays

The bacterial strains S. parauberis KSP28, *S. parauberis* SPOF3K, *P. damselae* FP2244, *A. sabnonicida*, *P. halotolerans* and *L. garvieae* FP MP5245, *P. aeruginosa*, *E. coli*, *E. cloacae*, *E. hormaechei*, *A. salmonicida*, *S. agalactiae*, *V. parahaemolyticus*, and *V. alginolyticus* were provided by National Fisheries Research and Development Institute, Korea. The vancomycin-resistant strains of E. faecium bacteria G1, G4, G7 and G13, and S. aureus, were provided by Ruijin Hospital, Shanghai Jiao Tong University School of Medicine. *E. piscicida* TH1, provided by the Chinese Academy of Tropical Agricultural Sciences, was also used in the antibacterial experiments.

The minimal inhibitory concentrations (MICs) for the compounds were determined using the 0.5 McFarland standard method. Mueller–Hinton II broth (cation-adjusted, BD 212322) was used for the bacterial culture. The compounds were generally dissolved in DMSO, to a concentration of 2 mM, to create stock solutions. All the samples were diluted in culture broth to an initial concentration of 100 µM. Serial 1:1 dilutions were then performed using culture broth, to achieve concentrations ranging from 100 µM to 0.24 µM. A total of 5 µL of each dilution was distributed into 96-well plates, along with sterile controls, growth controls (containing culture broth and DMSO without compounds), and positive controls (containing culture broth with antibiotics). All wells except the sterile control well were inoculated with 95 µL of an exponential-phase bacterial suspension (approximately 10^5^ CFU/well). The plates were incubated at 37 °C for 12 h. The MIC values were defined as the lowest concentration that completely inhibited bacterial growth. All the MIC values were interpreted in accordance with the Clinical and Laboratory Standards Institute (CLSI) guidelines. The MIC in molar concentration was ultimately converted to mass concentration, based on the molecular weight of each compound.

### 3.8. Cell Culture and Cell Viability

Rat liver Ac2F cells were obtained from the American Type Culture Collection (ATCC, Rockville, MD, USA). The cells were grown in Dulbecco’s modified Eagle medium (DMEM), containing 2 mM L-glutamine, 100 mg/mL streptomycin, 2.5 mg/L amphotericin B, and 10% heat-inactivated fetal bovine serum (FBS), and they were maintained in a humidified atmosphere containing 5% CO_2_ at 37 °C. The cells were seeded in 96-well culture plates, cultured for 12 h, and then treated with various concentrations of samples for 24 h. The cell viabilities were evaluated using a water-soluble tetrazolium (CCK-8) reagent, which was added to each well (10 mL) and incubated at 37 °C for 1 h. The absorbances were read using a microplate absorbance reader at a wavelength of 450 nm.

### 3.9. Luciferase Transactivation Assays

For the luciferase assays, the 3 × AOX-TK-luciferase reporter plasmid, containing 3 copies of the PPRE in the acyl CoA oxidase promoter, was a gift from Dr. Chistopher K. Glass (University of California at San Diego, La Jolla, CA, USA). The pcDNA3 expression vector and full-length human PPAR-α, PPAR-β, or PPAR-γ expression vector (pFlag-PPAR-α, pFlag-PPAR-β, or pFlag-PPAR-γ) were gifts from Dr. Chatterjee (the University of Cambridge, Addenbrooke’s Hospital at Cambridge, UK). For the experiment, 1 μg of 3 × AOX-TK-luciferase reporter plasmid was transfected into Ac2F cells in a 48-well plate (1 × 10^5^ cells/well) with 0.1 μg of effector plasmids, including pcDNA3 or pFlag-PPAR-α/β/γ, by using Lipofectamine™ 2000, as per the manufacturer’s instructions. After transfection for 6 h, the conditioned medium was removed and replaced with serum-free medium, and the compounds were added. After an additional incubation for 7 h, the cells were washed with PBS and assayed with the Luciferase Assay System (Promega, Madison, WI, USA). Luciferase activity was measured using a GloMax-Multi Microplate Multimode Reader (EnVision, PerkinElmer, Pontyclun UK).

### 3.10. Statistical Analysis

Each experiment was repeated at least three times. The experimental data were analyzed using one-way analysis of variance (ANOVA), and are presented as the mean ± standard deviation after assessment.

## 4. Conclusions

In summary, two previously unreported polycyclic diterpenoids (**1** and **2**), together with two structural analogues (**3** and **4**), were isolated and characterized from the soft coral *S. tortuosum*. Structurally, the new compounds **1** and **2** represent the first example of cembrane-derived tricyclic diterpenes belonging to the sarcophytin family to be isolated from *S. tortuosum*, which expands the chemical diversity of diterpenes from soft coral of this species. To the best of our knowledge, only ten members have been reported from the soft corals of the *Sarcophyton* genus and their predator sea slug *Phyllodesmium longicirrum* [2,15]. The stereochemistry of the new compounds was unambiguously determined using a combination of X-ray diffraction analysis, the QM-NMR method, and a TDDFT-ECD calculation. A plausible biosynthetic pathway for compounds **1**–**4** was proposed by carrying out an interesting transannular Diels–Alder cycloaddition. The intramolecular 4+2 cycloaddition reactions occurring in compounds **1** and **2** exhibited significant differences, manifesting in different diene positions, which resulted in the carbomethoxy group being attached to the C-10 position of compound **1** and the C-4a position of compound **2**, respectively. A study by Pierre and coworkers achieved an asymmetric total synthesis of (+)-chatancin via a transannular Diels–Alder reaction, demonstrating the rationality of the above proposed biosynthetic pathway [13]. In bioassays, the new compound **1** exhibited antibacterial activities against the fish pathogens *S. parauberis* KSP28, oxytetracycline-resistant *S. parauberis*, and *P. damselae* FP2244, while compound **3** showed selective PPAR-β agonist activity. These intriguing findings highlight the potential of sarcophytin-like tricyclic diterpenes as leading compounds for the treatment of microbial infections and metabolic diseases.

## Figures and Tables

**Table 1:** ^1^H (600 MHz) and ^13^C NMR (150 MHz) data of compounds **1** and **2** in CDCl_3_.

No.	1		2	
	*δ*_H_, Mult (*J* in Hz)	*δ*_C_, Type	*δ*_H_, Mult (*J* in Hz)	*δ*_C_, Type
1		98.4 qC	1.82 dd (13.1, 6.5)	31.8 CH_2_
			1.46 dd (13.1, 8.3)	
2	1.56 ovl	47.0 CH	2.18 m	46.1 CH
3	1.59 ovl	27.6 CH_2_		106.4 qC
	1.73 m			
4	3.53 m	77.0 CH	1.44 d (12.8)	45.6 CH_2_
			3.11 d (12.8)	
4a		41.6 qC		58.7 qC
4b	1.60 ovl	43.8 CH	2.91 d (13.0)	58.3 CH
5	1.52 m, 0.70 m	28.6 CH_2_		197.9 qC
6	0.98 m, 1.66 ovl	34.6 CH_2_	5.86 s	127.5 CH
7	1.64 ovl	29.8 CH		161.5 qC
8	1.25 m	42.3 CH_2_	2.25 m	36.9 CH_2_
	2.05 d (12.7)		2.51 dd (17.7, 5.0)	
8a		76.2 qC	3.02 m	32.8 CH
9	7.27 s	145.1 CH	5.89 s	135.3 CH
10		134.8 qC		134.2 qC
10a	2.79 s	51.0 CH		81.5 qC
11	2.30 m	25.4 CH	1.98 m	26.9 CH
12	0.93 d (7.0)	18.3 CH_3_	0.88 d (6.7)	17.9 CH_3_
13	0.93 d (7.0)	23.1 CH_3_	0.9 d (6.7)	21.5 CH_3_
14	0.85 s	20.9 CH_3_		175.3 qC
15	1.00 d (6.3)	22.4 CH_3_	1.96 s	24.5 CH_3_
16		165.4 qC	1.86 s	19.8 CH_3_
17	3.80 s	52.2 CH_3_	3.71 s	52.7 CH_3_

**Table 2:** Antibacterial activities of compound **1** and antibiotics ^a^.

Compounds	MIC (μg/mL)		
	*S. parauberis*	*S. parauberis* SPOF3K	*P. damselae* FP2244
**1**	9.10	9.10	18.21
Tetracycline	3.01	>24.00	0.02
Oxytetracycline	1.55	12.42	0.02
Levofloxacin	1.24	1.24	0.02
Ampicillin	4.64	0.58	0.02

^a^ Tetracycline, oxytetracycline, levofloxacin, and ampicillin were used as positive controls.

## Data Availability

The data that support the findings of this study are available in the Supplementary Material of this article.

## Supplementary Materials

The following supporting information can be downloaded at https://www.mdpi.com/article/10.3390/md22120553/s1: Figures S1–S20: NMR, HRESIMS, IR, UV and CD data of compounds **1**–**2**. Figure S21. Structures of isomers of compound **2**. Table S1: Crystal data for compound **1**. Tables S2 and S3: Quantum chemical calculations of NMR shifts for compound **2**.
