# Supplementary material for: Two New Diterpenoids Formed by Transannular Diels–Alder Cycloaddition from the Soft Coral Sarcophyton tortuosum, and Their Antibacterial and PPAR-β Agonist Activities
Source: Mar Drugs. 2024 Dec 10;22(12):553. doi: 10.3390/md22120553 (PMC11678790; doi:10.3390/md22120553)
Supplement: Supplementary file 1 [file marinedrugs-22-00553-s001.zip › marinedrugs-3352580-supplementary.pdf]

Supporting Information

Two New Diterpenoids Formed by Transannular Diels–Alder Cycloaddition from the Soft Coral *Sarcophyton tortuosum*, and Their Antibacterial and PPAR-β Agonist Activities

Min Sun [1,2,†], Songwei Li [3,†], Jianang Zeng [2], Yuewei Guo [2,3,*], Changyun Wang [1,4,*] and Mingzhi Su [2,*]

[1] MOE Key Laboratory of Marine Drugs and Key Laboratory of Evolution and Marine Biodiversity, Institute of Evolution & Marine Biodiversity, School of Medicine and Pharmacy, Ocean University of China, Qingdao 266003, China; 21220813006@stu.ouc.edu.cn
[2] Shandong Laboratory of Yantai Drug Discovery, Bohai Rim Advanced Research Institute for Drug Discovery, Yantai 264117, China; zjianang05@163.com
[3] School of Medicine, Shanghai University, Shanghai 200444, China; songweili@shu.edu.cn
[4] Laboratory for Marine Drugs and Bioproducts, Qingdao Marine Science and Technology Center, Qingdao 266237, China
* Correspondence: ywguo@simm.ac.cn (Y.G.); changyun@ouc.edu.cn (C.W.); mzsu@baridd.ac.cn (M.S.)
† These authors contributed equally to this work.

CONTENT

S1. Spectra of compound **1** 3

Table S1. Crystal data for compound **1** 3

Figure S1. ^{1}H NMR spectrum (600 MHz) of compound **1** in $CDCl_3$........ 4

Figure S2. ^{13}C NMR spectrum (150 MHz) of compound **1** in $CDCl_3$. 5

Figure S3. DEPT spectrum 150 MHz) of compound **1** in $CDCl_3$ 5

Figure S4. HSQC spectrum (600 MHz) of compound **1** in $CDCl_3$ 6

Figure S5. HMBC spectrum (600 MHz) of compound **1** in $CDCl_3$ 8

Figure S6. ^{1}H－^{1}H COSY spectrum (600 MHz) of compound **1** in $CDCl_3$ 9

Figure S7. NOESY spectrum (600 MHz) of compound **1** in $CDCl_3$........ 10

Figure S8. HR-ESI-MS spectrum of compound **1** 10

Figure S9. IR spectrum of compound **1** 11

Figure S10. UV and CD spectrum of compound **1** 11

S2. Spectra of compound **2** 12

Figure S11. ^{1}H NMR spectrum (600 MHz) of compound **2** in $CDCl_3$........ 12

Figure S12. ^{13}C NMR spectrum (150MHz) of compound **2** in $CDCl_3$ 13

Figure S13. DEPT spectrum (150 MHz) of compound **2** in $CDCl_3$........ 13

Figure S14. HSQC spectrum (600 MHz) of compound **2** in $CDCl_3$ 14

Figure S15. HMBC spectrum (600 MHz) of compound **2** in $CDCl_3$ 15

Figure S16. ^{1}H－^{1}H COSY spectrum (600 MHz) of compound **2** in $CDCl_3$ 16

Figure S17. NOESY spectrum (600 MHz) of compound **2** in $CDCl_3$........ 17

Figure S18. HR-ESI-MS spectrum of compound **2** 18

Figure S19. IR spectrum of compound **2** 19

Figure S20. UV and CD spectrum of compound **2** 19

S3. Quantum chemical calculations of NMR shifts for compound **2** 20

S3.1.Structures of isomers studied for compound **2** 20

Figure S21. Structures of isomers of compound **2** 20

S3.2. Isotropic magnetic shielding constants of compound **2** 20

Table S2. Boltzmann-averaged GIAO isotropic magnetic shielding constants (σ) of compound **2** calculated at the PCM/mPW1PW91/6-31G*//B3LYP/6-311G(d,p) level of theory 20

S3.3. DP4+ results of compound **2** 21

Table S3. DP4+ results obtained using experimental data of compound **2** versus isomers **2a**, **2b**, **2c** and **2d** 21

S1. Spectra of compound 1

Table S1. Crystal data for compound 1.

Identification code	cu_20240026_0m
Empirical formula	$C_{21}H_{34}O_6$
Formula weight	382.48
Temperature/K	150.00
Crystal system	monoclinic
Space group	C2
a/Å	19.2786(5)
b/Å	11.3201(3)
c/Å	10.4695(3)
α/°	90
β/°	115.5450(10)
γ/°	90
Volume/Å^3	2061.47(10)
Z	4
ρ_{calc}g/cm^3	1.232
μ/mm^{-1}	0.725
F(000)	832.0
Crystal size/mm^3	0.2 × 0.15 × 0.04
Radiation	CuKα (λ = 1.54178)
2Θ range for data collection/°	10.17 to 141
Index ranges	-23 ≤ h ≤ 23, -13 ≤ k ≤ 13, -12 ≤ l ≤ 12
Reflections collected	31014
Independent reflections	3908 [R_{int} = 0.0656, R_{sigma} = 0.0341]
Data/restraints/parameters	3908/259/267
Goodness-of-fit on F^2	1.047
Final R indexes [I>=2σ (I)]	R_1 = 0.0331, wR_2 = 0.0810
Final R indexes [all data]	R_1 = 0.0348, wR_2 = 0.0827
Largest diff. peak/hole / e Å^{-3}	0.23/-0.22
Flack parameter	0.08(7)

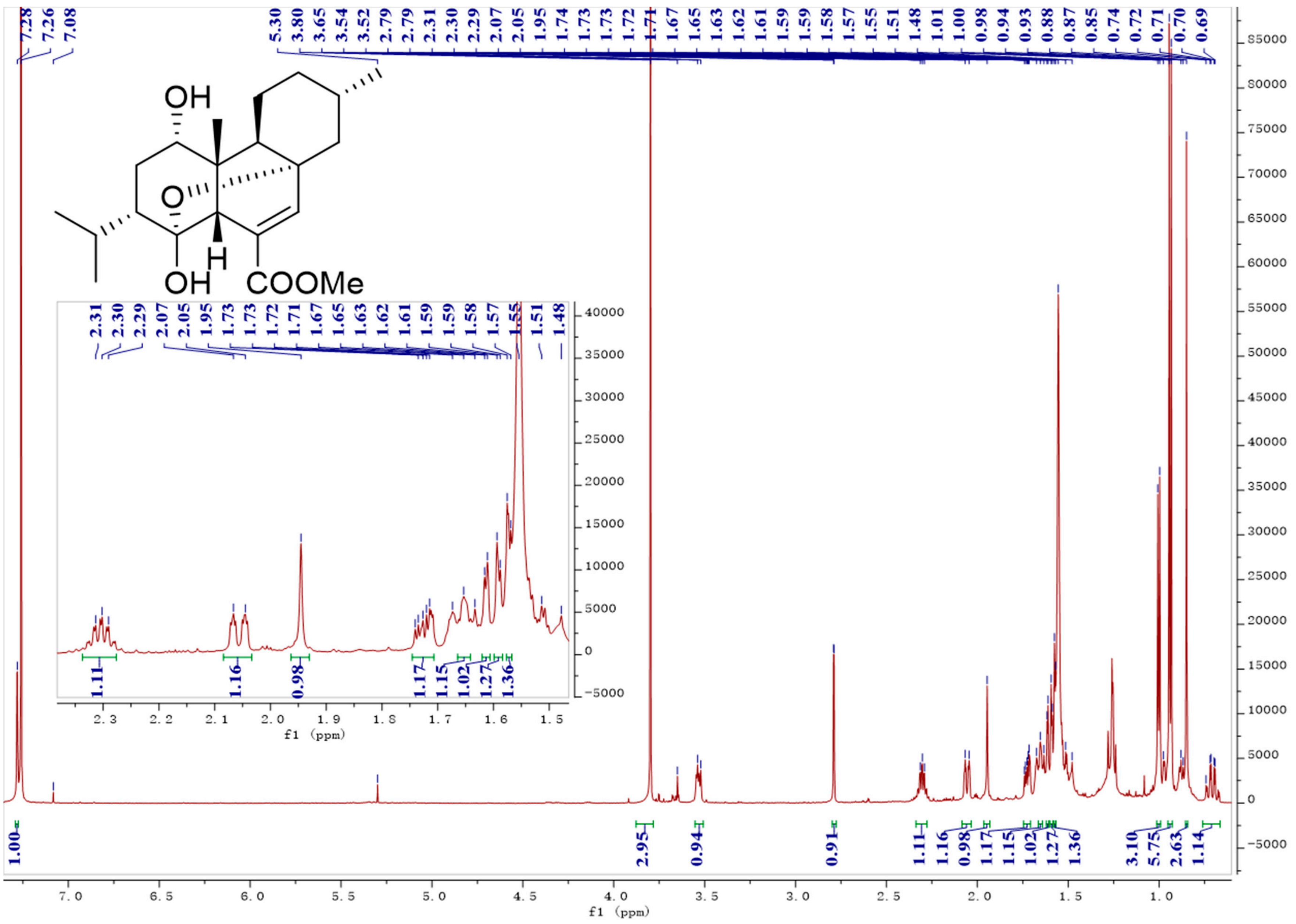

Figure S1. 1H NMR spectrum (600 MHz) of compound 1 in CDCl3.

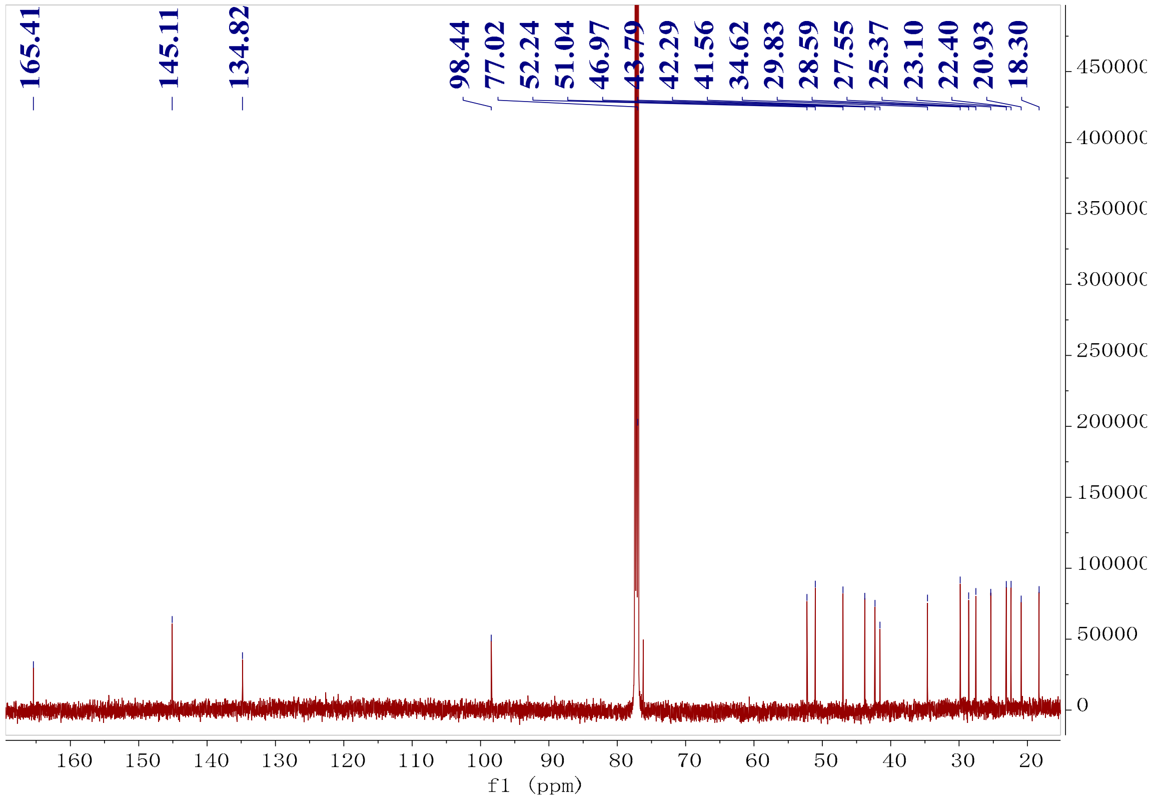

Figure S2. ^{13}C NMR spectrum (150 MHz) of compound 1 in $CDCl_3$.

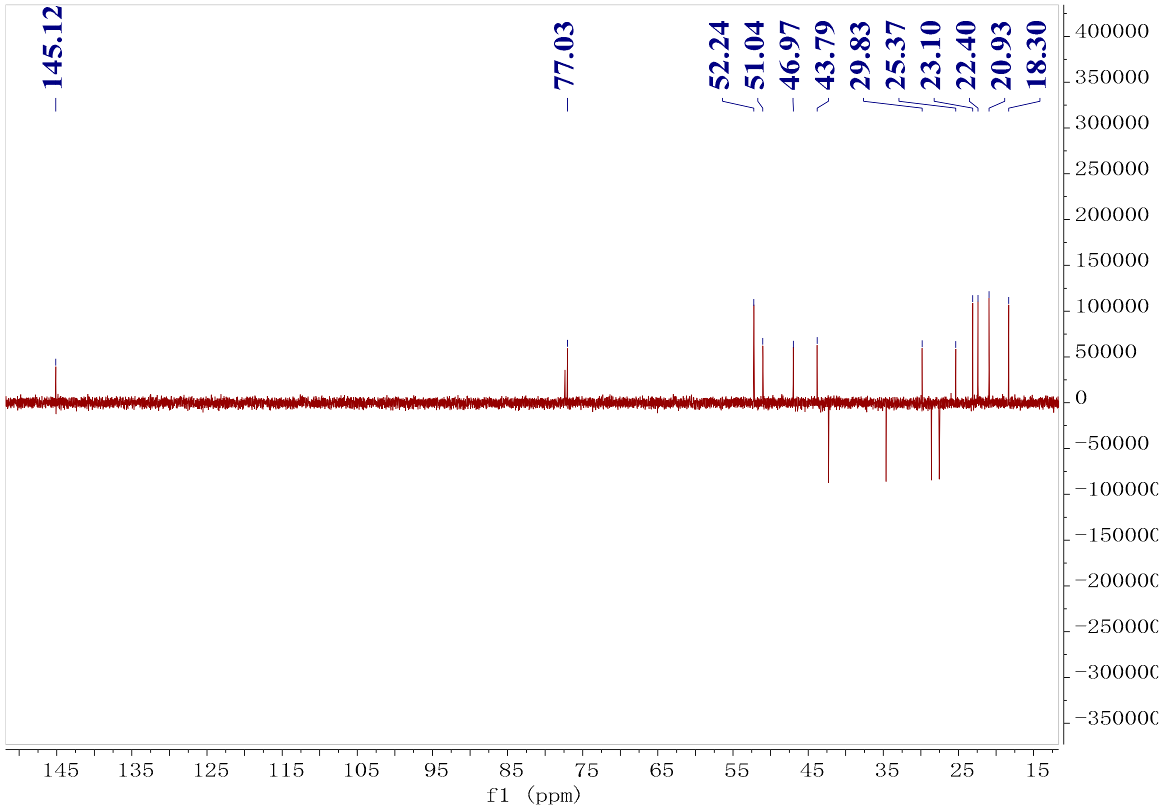

Figure S3. DEPT spectrum (150 MHz) of compound 1 in $CDCl_3$.

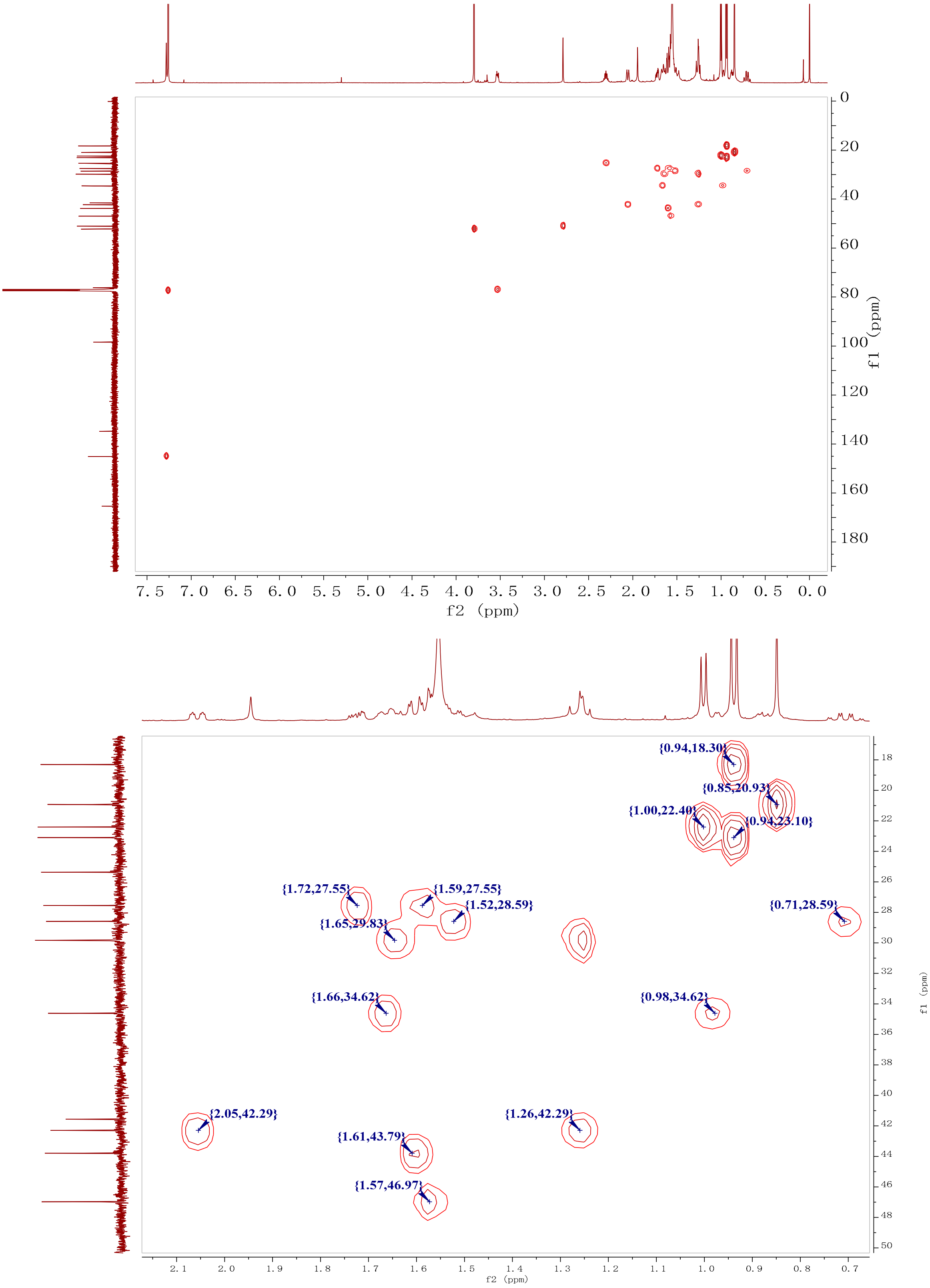

Figure S4. HSQC spectrum (600 MHz) of compound 1 in $CDCl_3$.

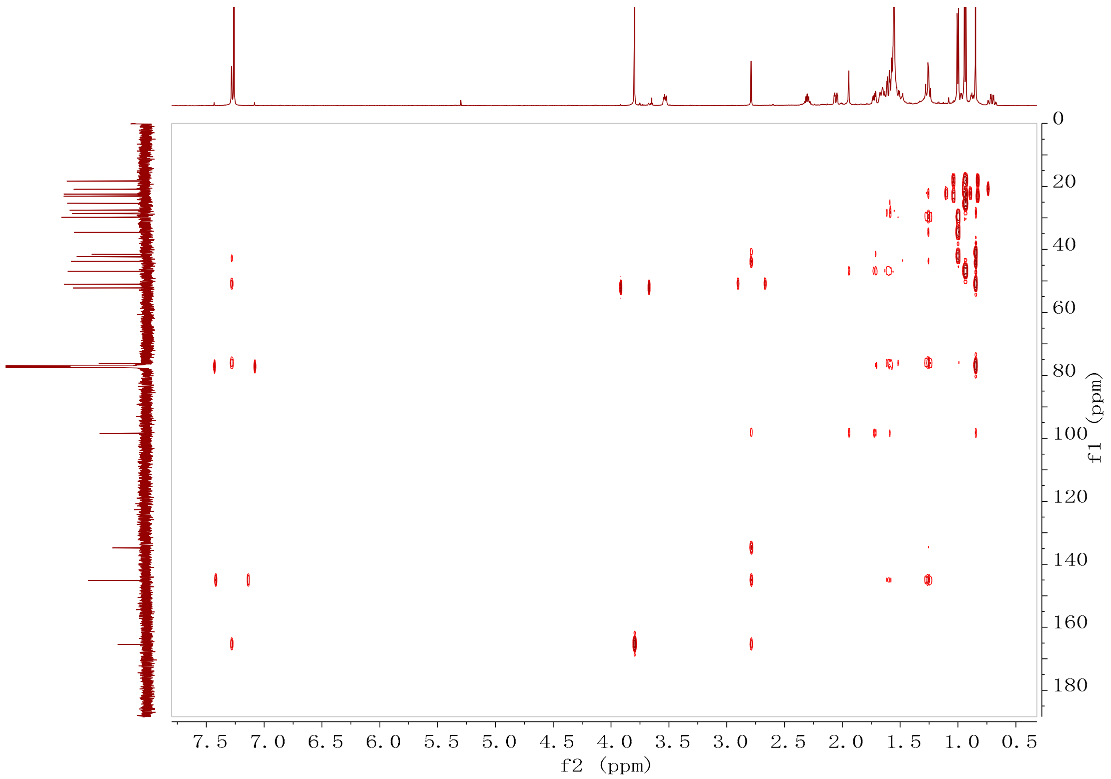
f2 (ppm)
f1 (ppm)

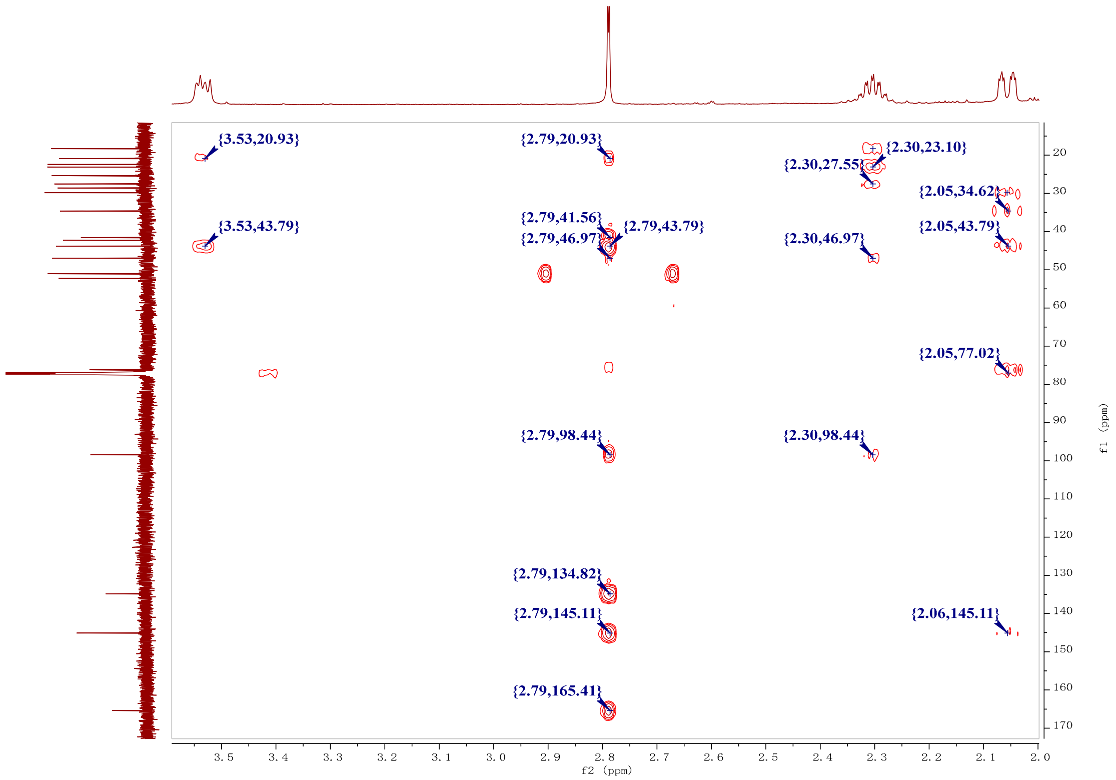
{3.53,20.93}
{2.79,20.93}
{2.30,23.10}
{2.30,27.55}
{2.05,34.62}
{2.79,41.56}
{2.79,43.79}
{3.53,43.79}
{2.79,46.97}
{2.30,46.97}
{2.05,43.79}
{2.05,77.02}
{2.79,98.44}
{2.30,98.44}
{2.79,134.82}
{2.79,145.11}
{2.06,145.11}
{2.79,165.41}
f2 (ppm)
f1 (ppm)

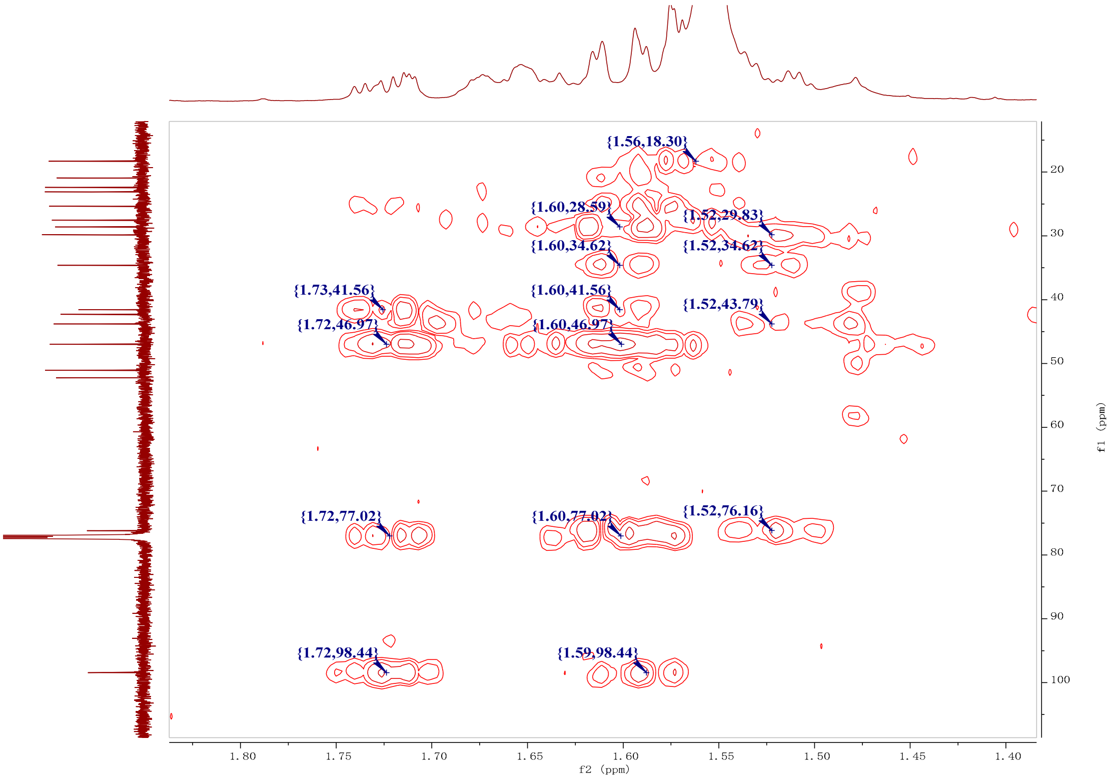

Figure S5. HMBC spectrum (600 MHz) of compound 1 in $CDCl_3$.

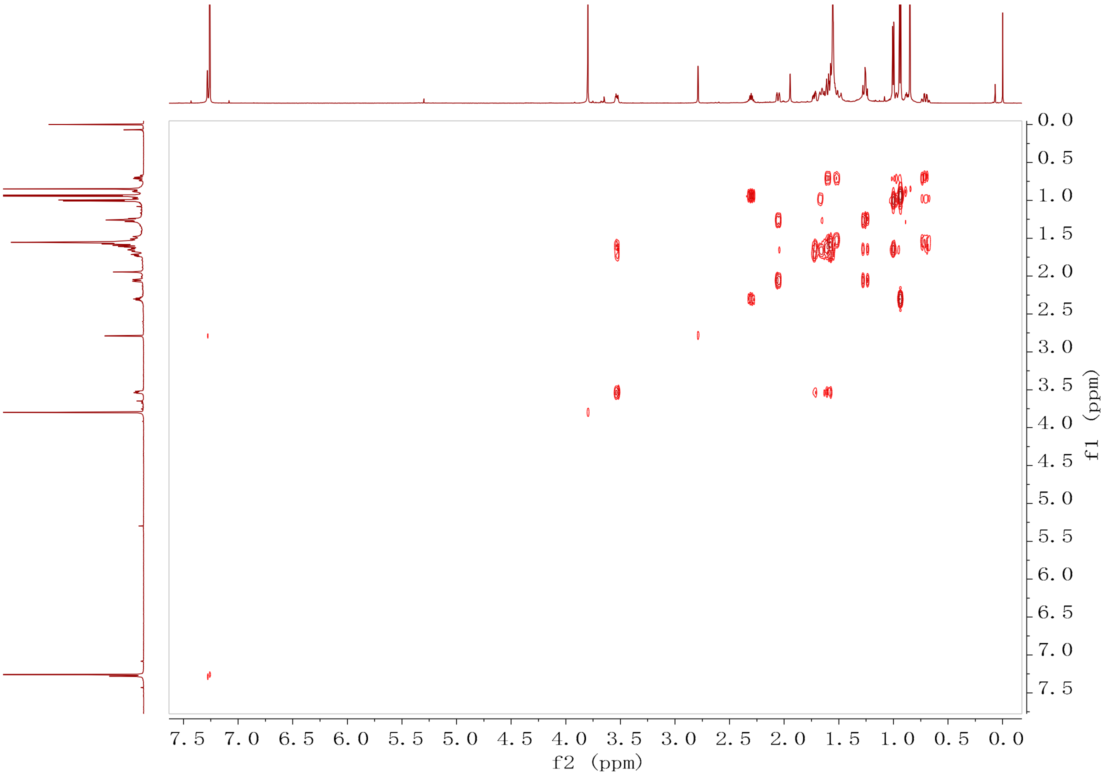

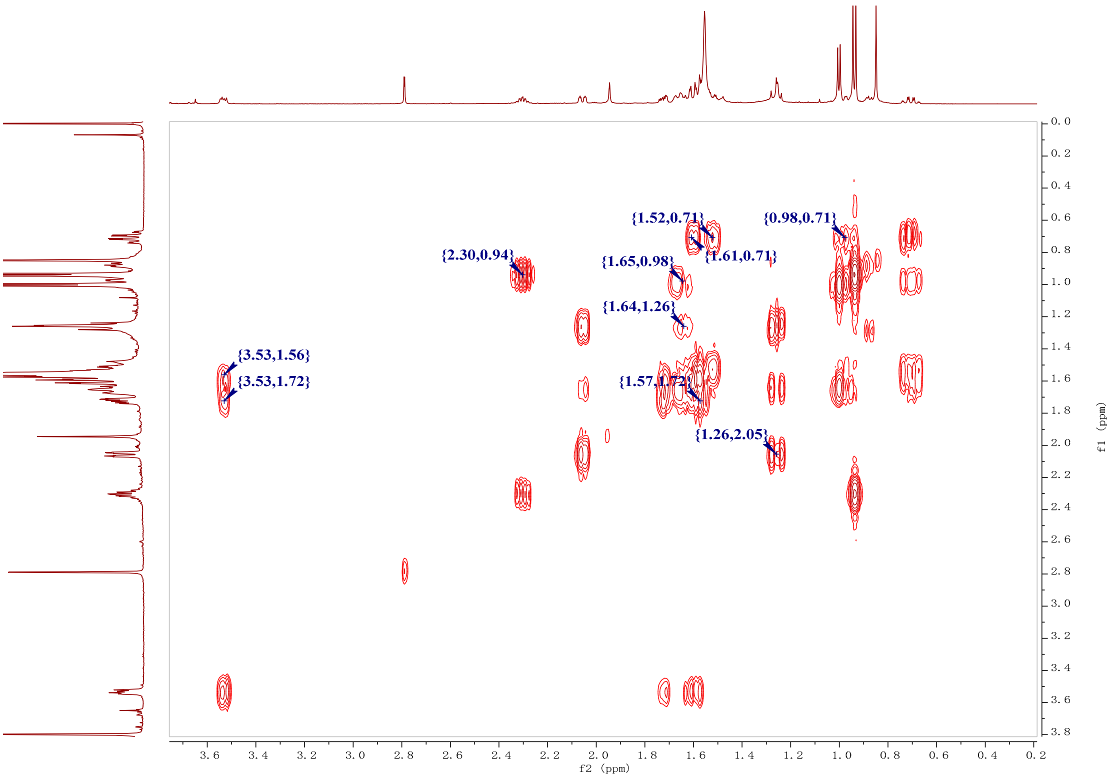

Figure S6. ^1H–^1H COSY spectrum (600 MHz) of compound 1 in $CDCl_3$.

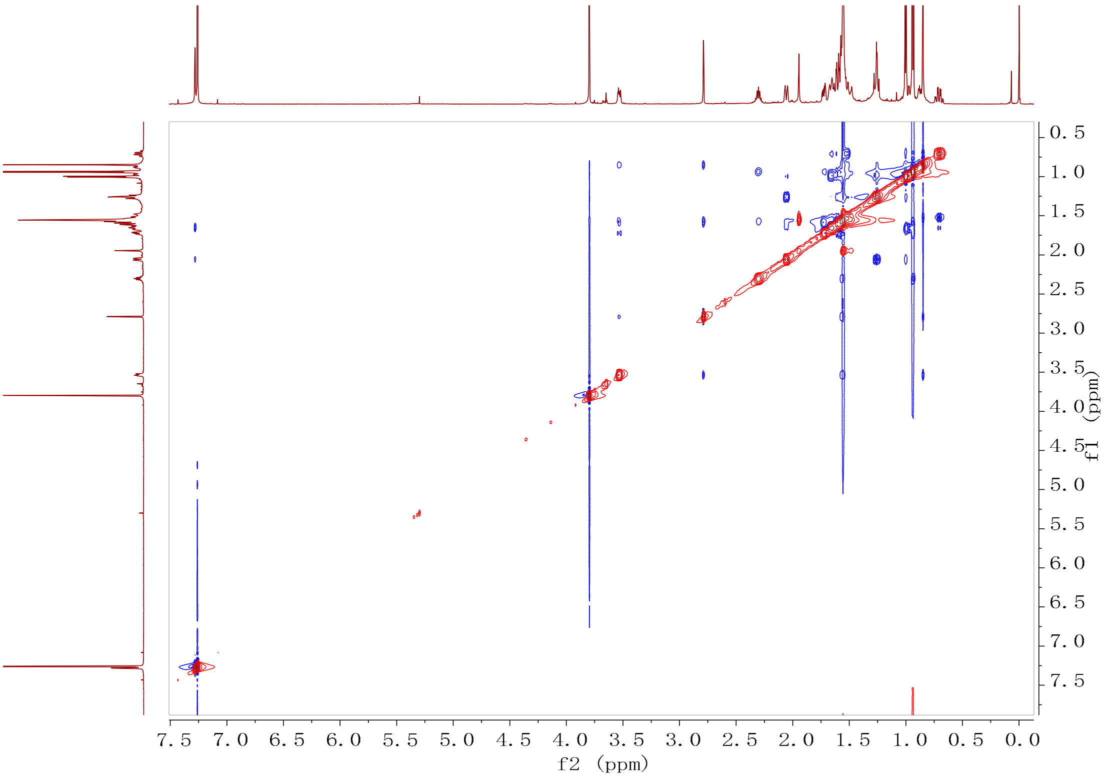

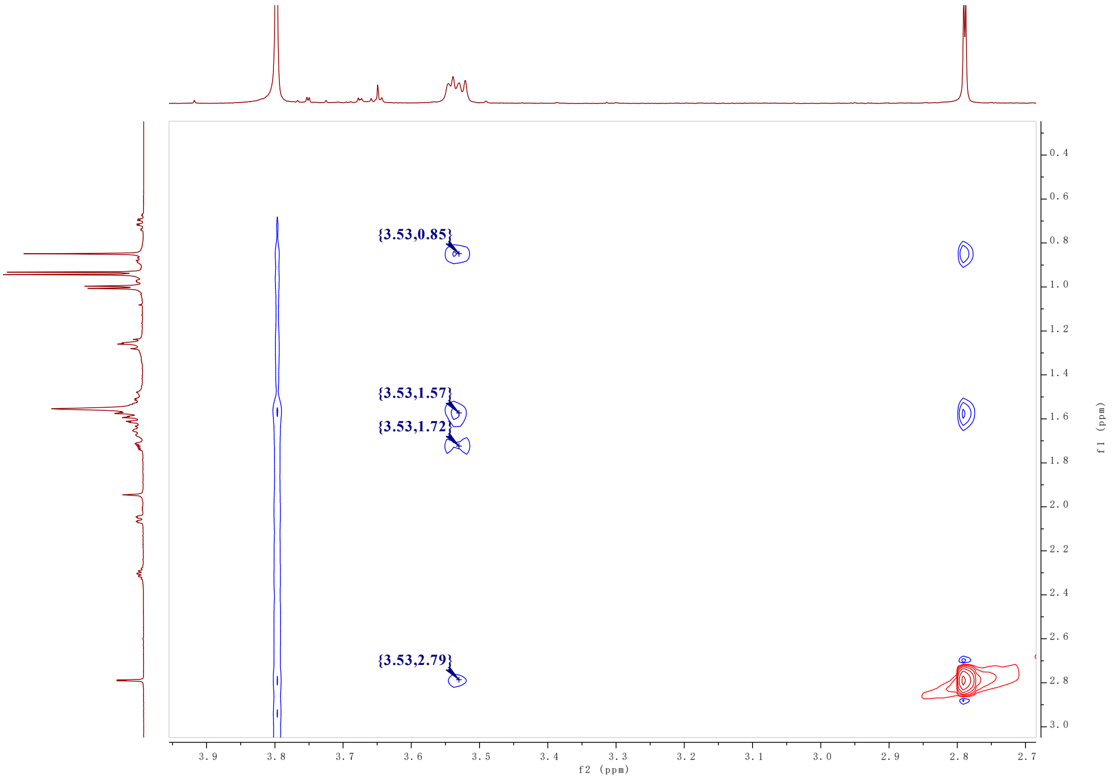

Figure S7. NOESY spectrum (600 MHz) of compound 1 in $CDCl_3$.

MS spectra

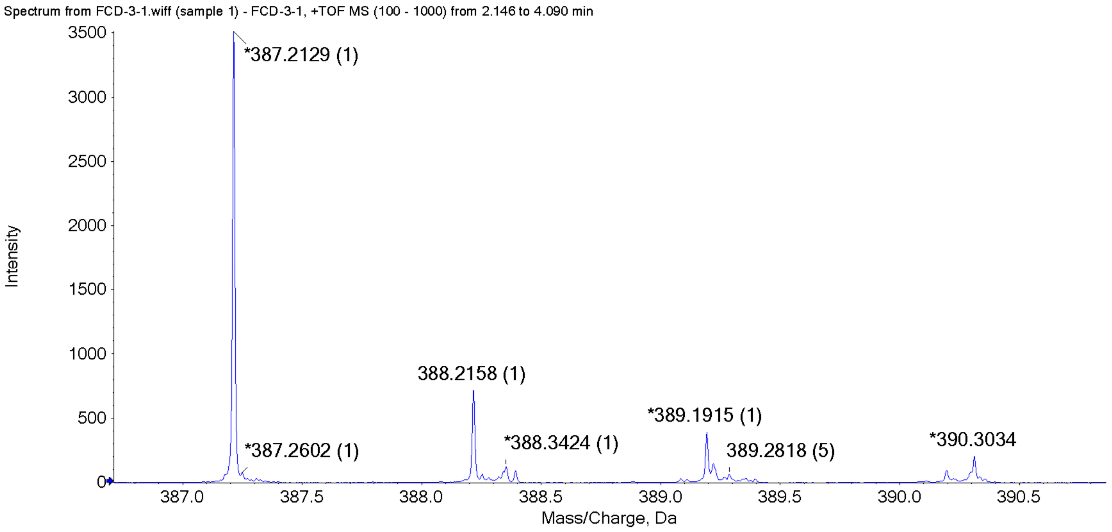

Formula Calculator Results

Measure m/z	Cal m/z	Error(mmu)	Error(ppm)	Ion Formula	Ion
387.2129	387.2147	-1.8	-4.8	$C_{21}H_{32}NaO_5$	$[M+Na]^+$

Figure S8. HR-ESI-MS spectrum of compound 1.

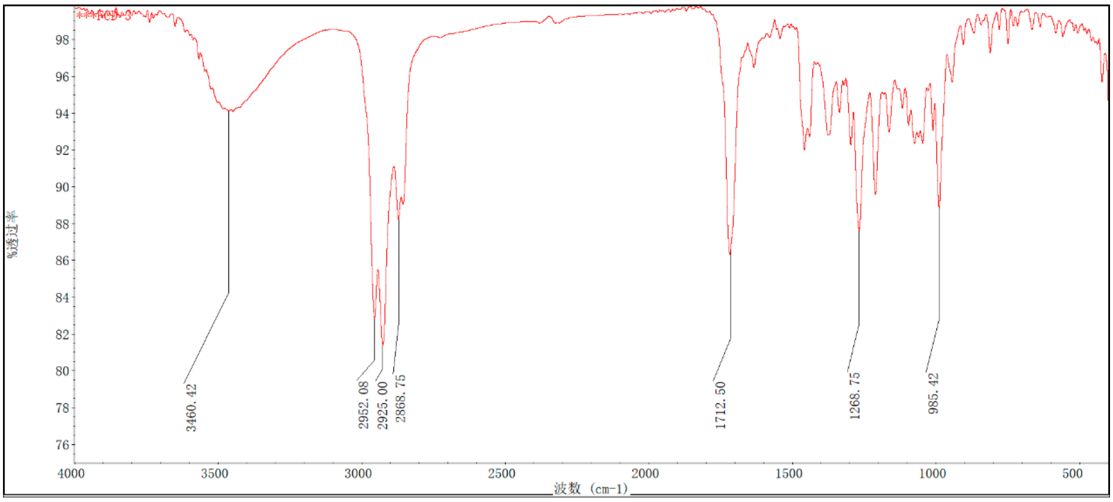

Figure S9. IR spectrum of compound 1.

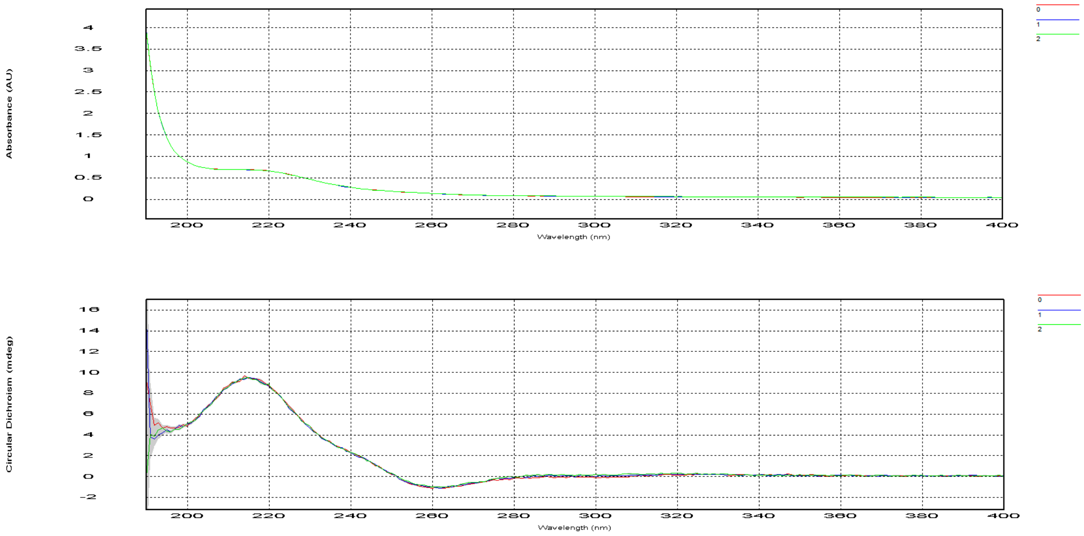

Figure S10. UV and CD spectrum of compound 1.

S2. Spectra of compound 2

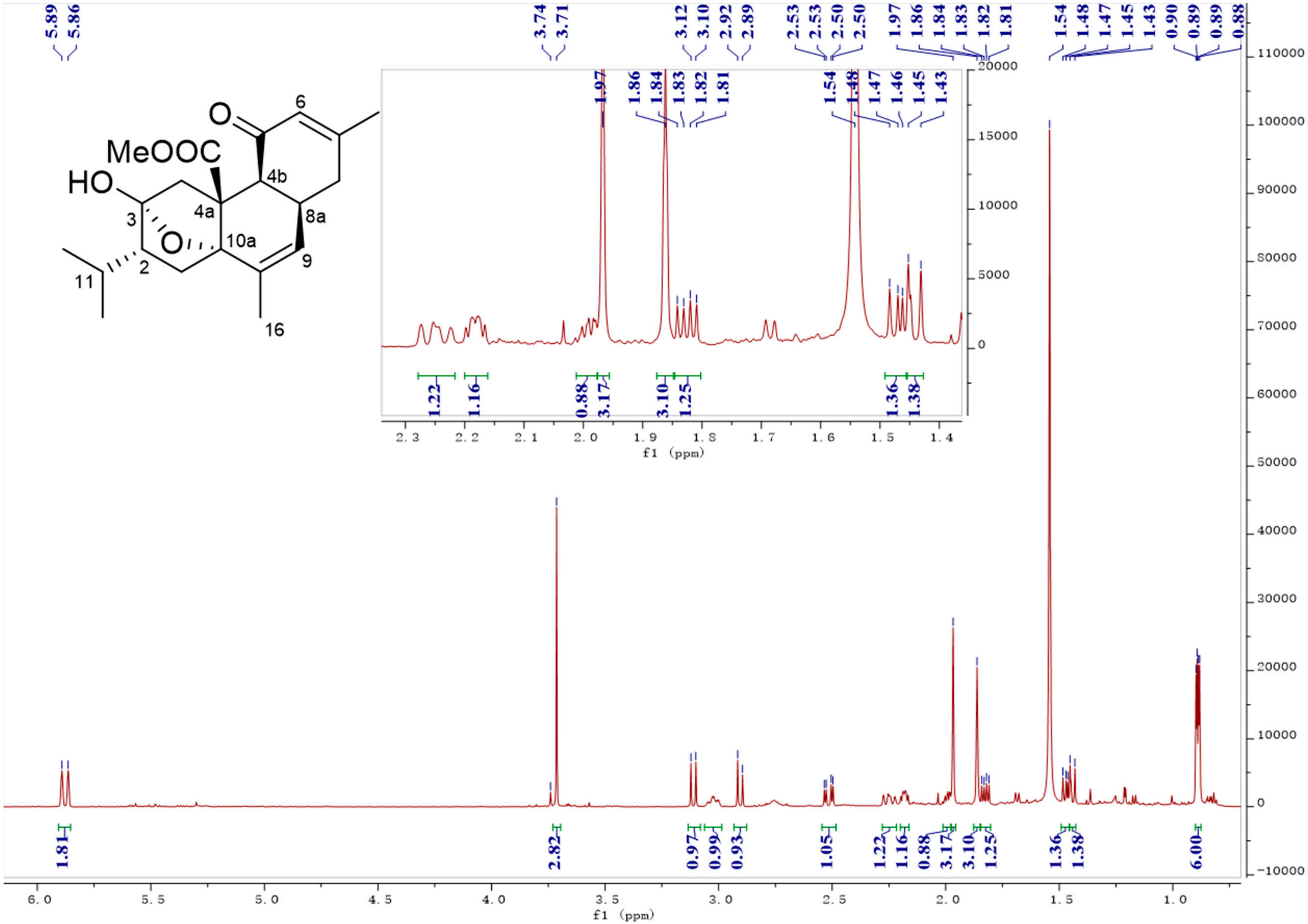

Figure S11. ^{1}H NMR spectrum (600 MHz) of compound 2 in $CDCl_3$.

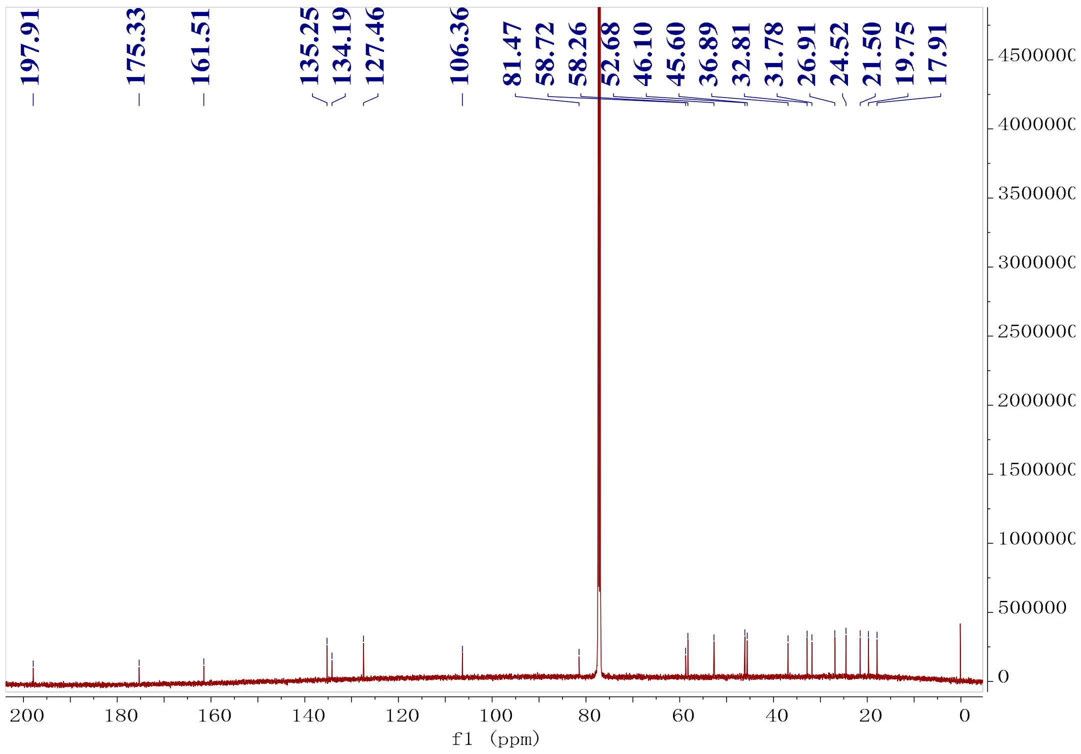

Figure S12. ^{13}C NMR spectrum (150MHz) of compound 2 in $CDCl_3$.

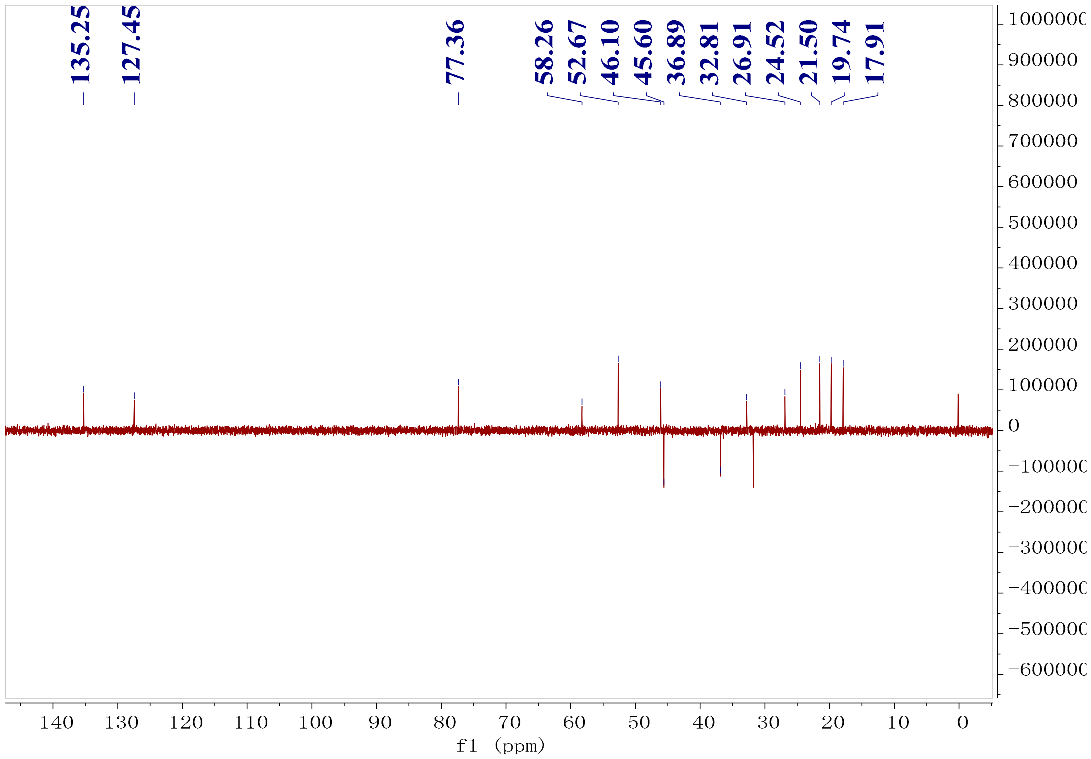

Figure S13. DEPT spectrum (150 MHz) of compound 2 in CDCl3.

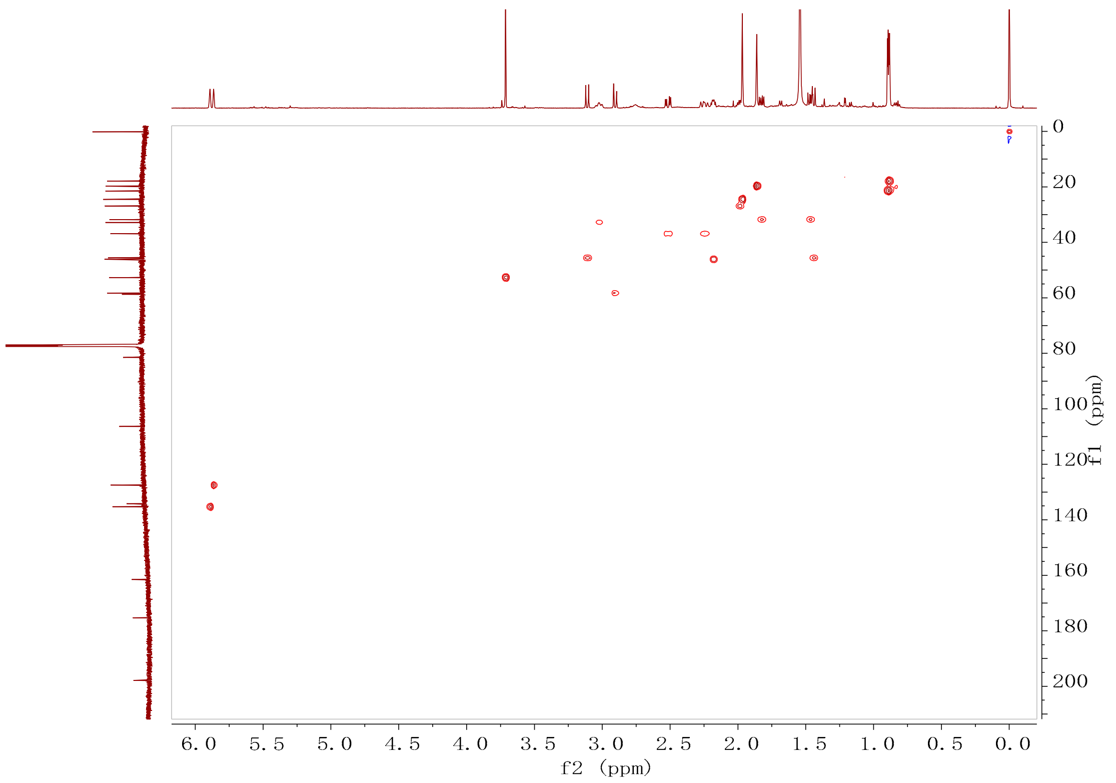

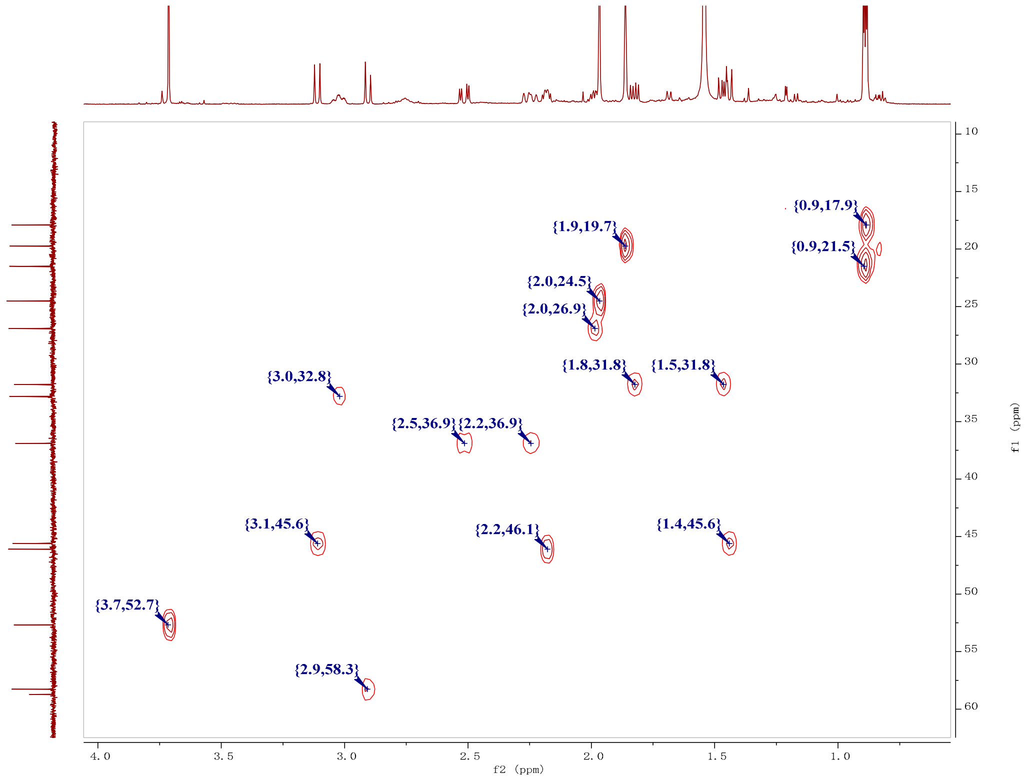

Figure S14. HSQC spectrum (600 MHz) of compound 2 in $CDCl_3$.

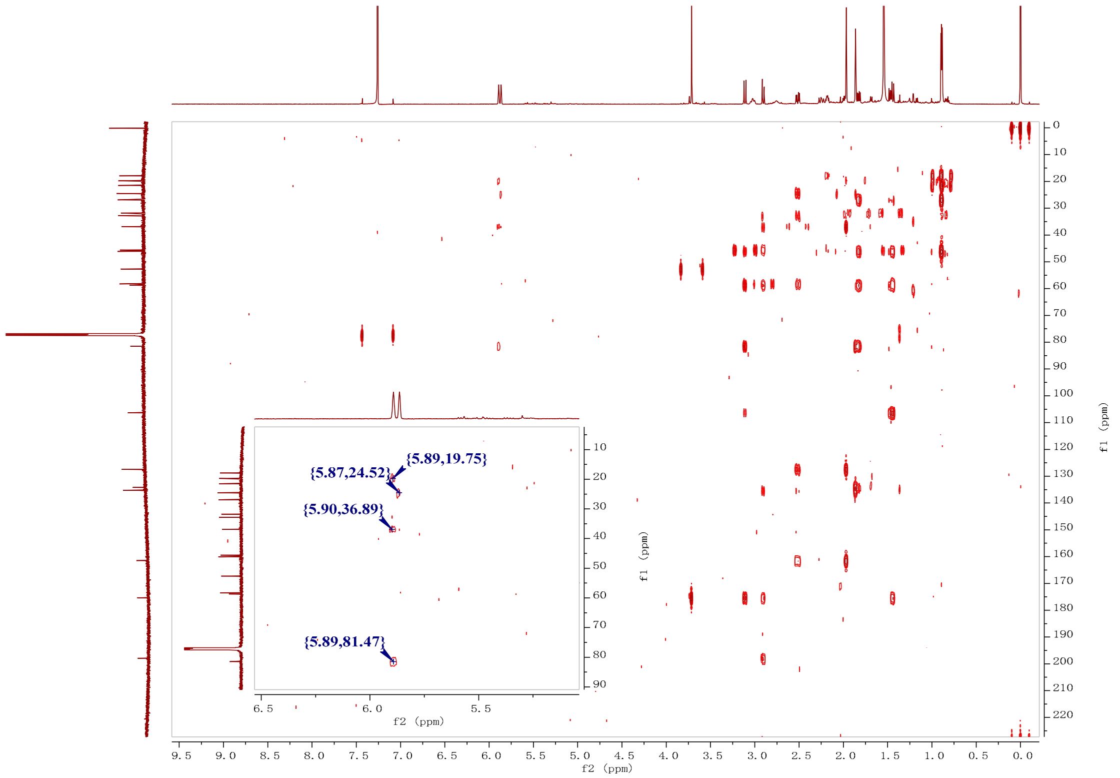

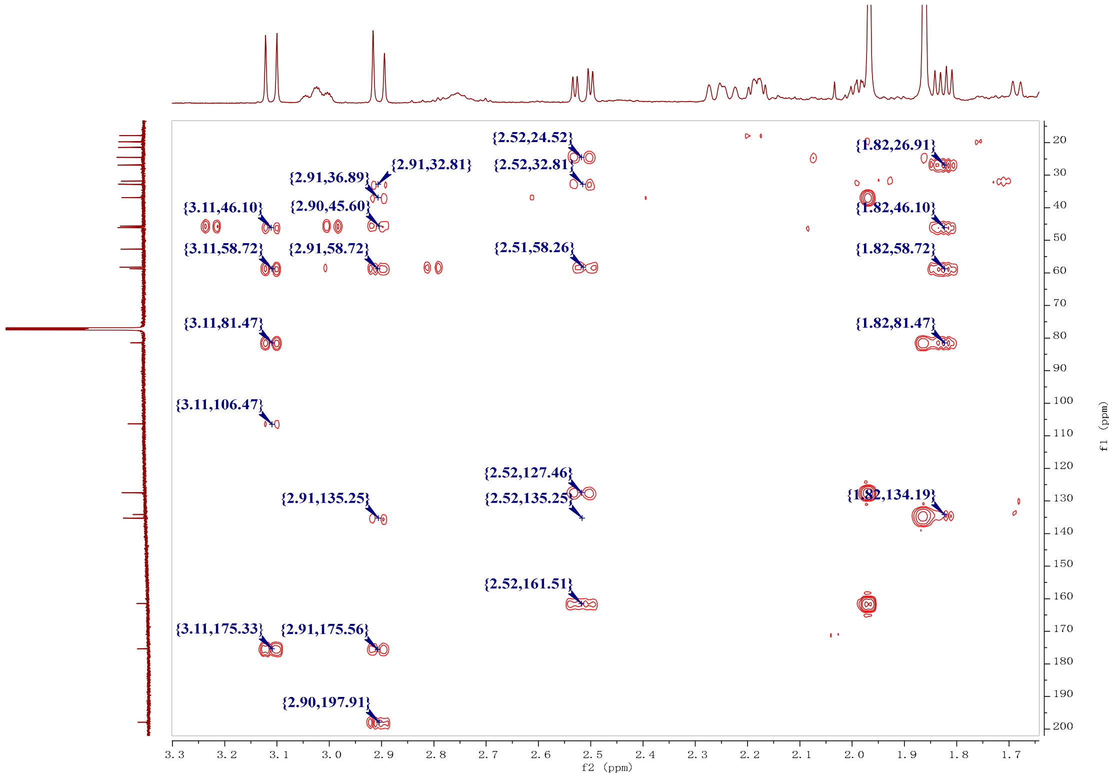

Figure S15. HMBC spectrum (600 MHz) of compound 2 in $CDCl_3$.

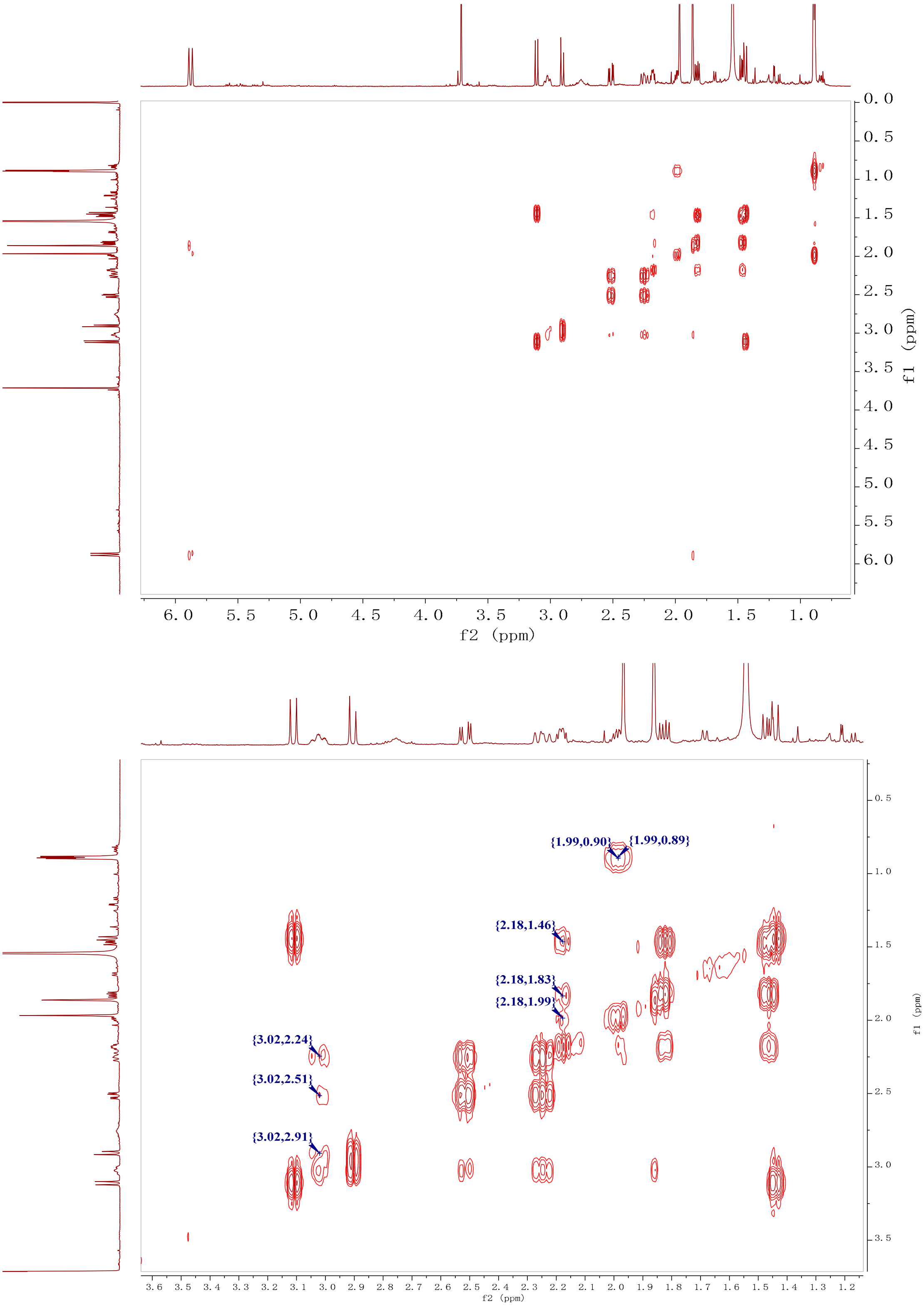

Figure S16. ^{1}H–^{1}H COSY spectrum (600 MHz) of compound 2 in $CDCl_3$.

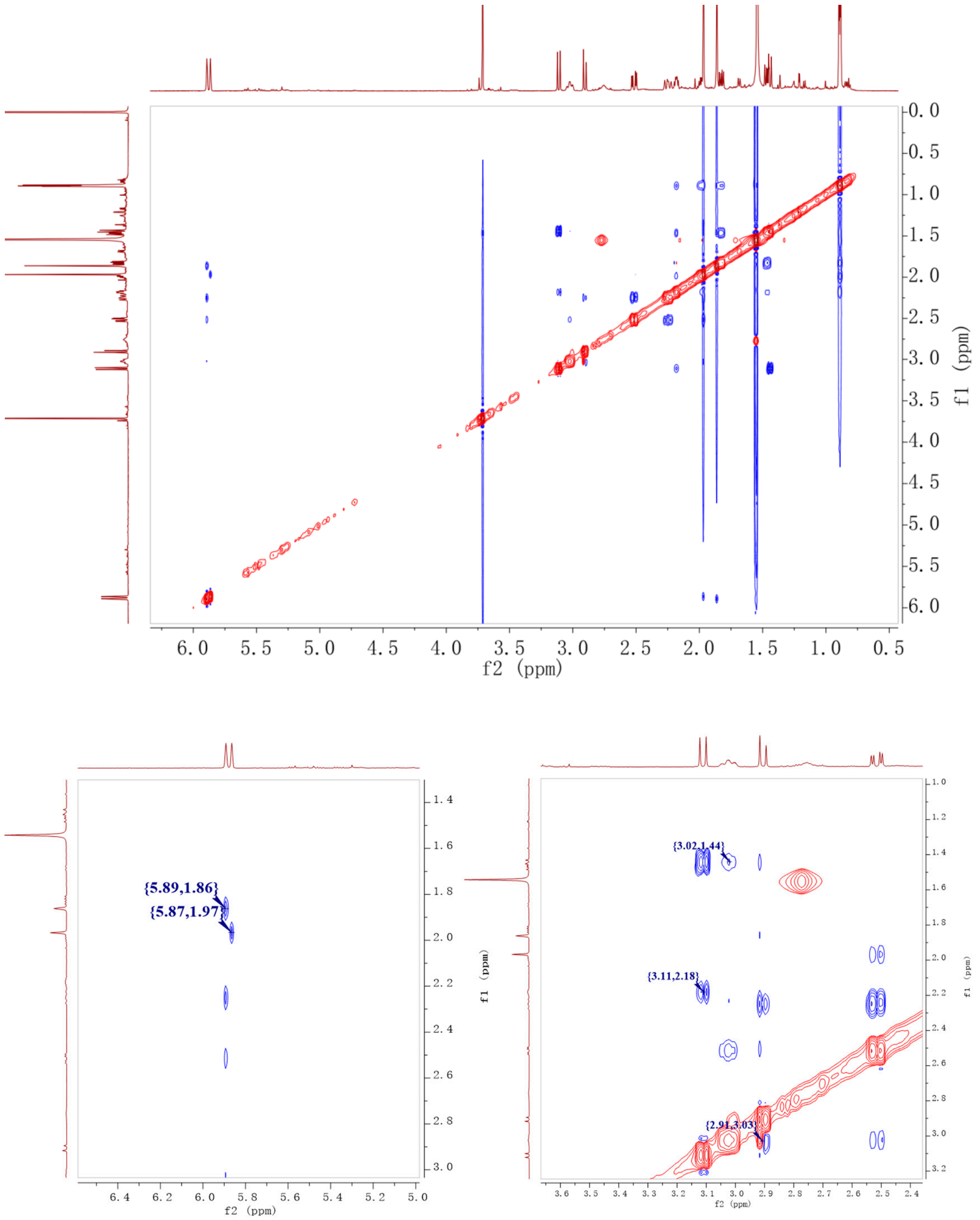

Figure S17. NOESY spectrum (600 MHz) of compound 2 in $CDCl_3$.

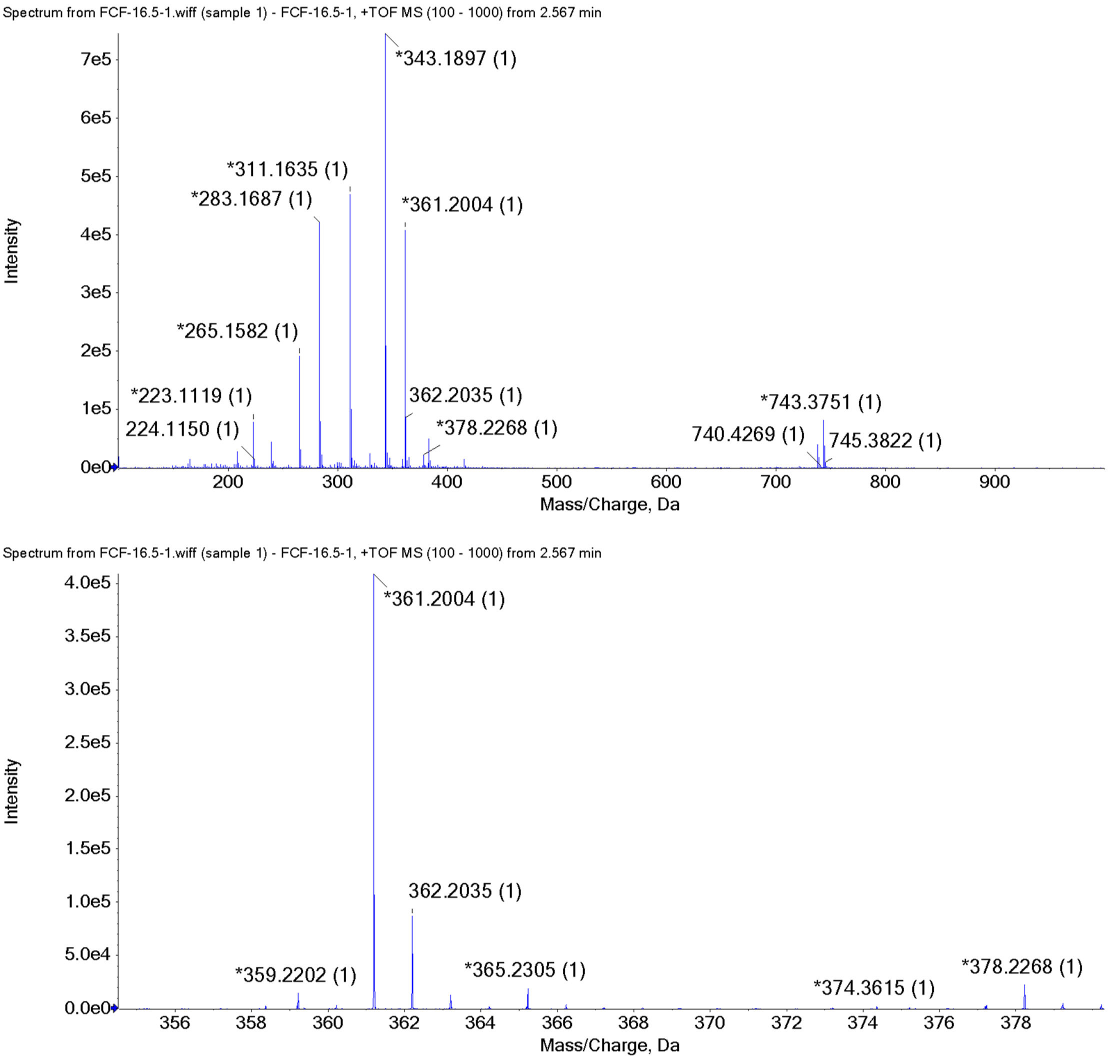

Formula Calculator Results

Measure m/z	Cal m/z	Error(mmu)	Error(ppm)	Ion Formula	Ion
361.2004	361.2010	-0.5	-1.5	$C_{21}H_{29}O_5$	$[M+H]^+$

Figure S18. HR-ESI-MS spectrum of compound 2.

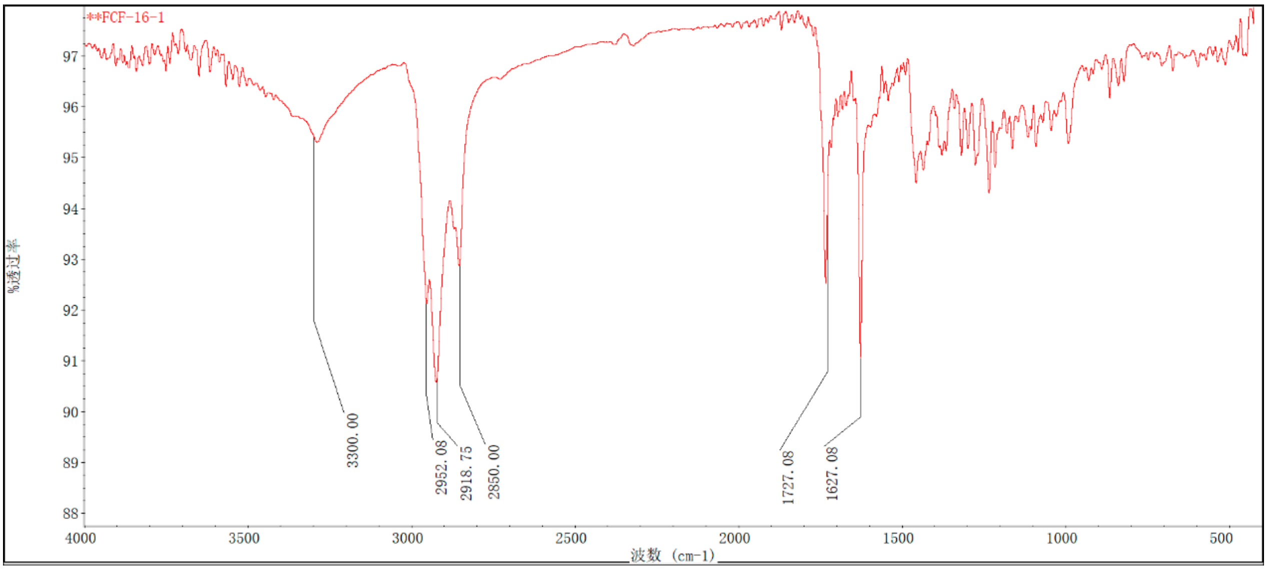

Figure S19. IR spectrum of compound 2.

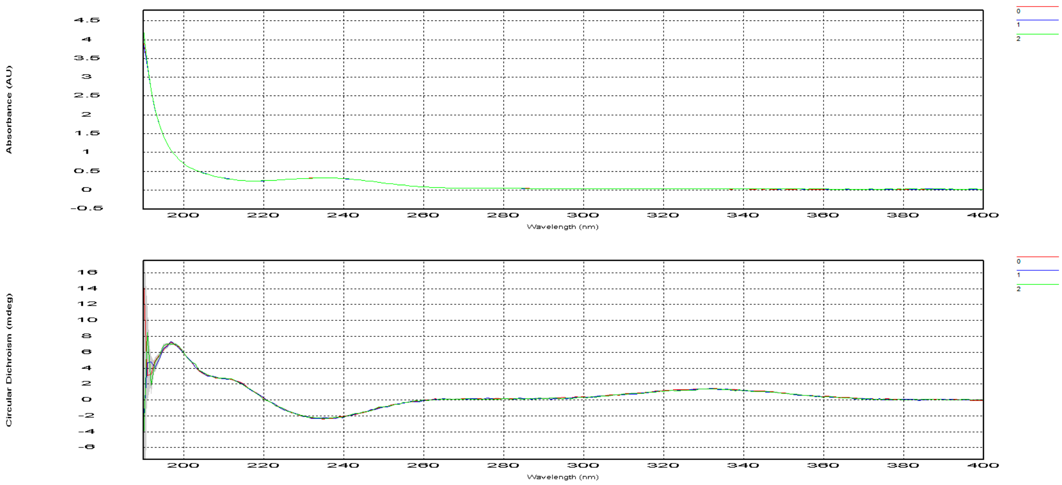

Figure S20. UV and CD spectrum of compound 2.

S3. Quantum chemical calculations of NMR shifts for compound 2

S3.1. Structures of isomers studied for compound 2.

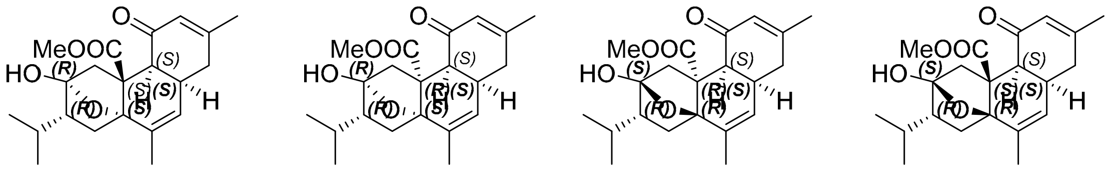

Isomer1 Isomer2 Isomer3 Isomer4

Figure S21. Structures of isomers of compound 2.

S3.2. Isotropic magnetic shielding constants of compound 2.

Table S2. Boltzmann-averaged GIAO isotropic magnetic shielding constants (σ) of compound 2 calculated at the PCM/mPW1PW91/6-31G*//B3LYP/6-311G(d,p) level of theory.

	Isotropic shielding constants			
Nuclei	2a	2b	2c	2d
C 1	151.7	143.1	148.7	142.5
C 2	134.7	135.2	131.1	131.0
C 3	75.4	73.2	74.8	72.6
C 4	135.9	138.4	147.9	144.5
C 5	-20.9	-20.3	-21.3	-19.3
C 6	54.6	53.7	52.6	54.2
C 7	12.3	13.4	16.5	14.5
C 8	151.9	149.3	148.4	149.9
C 9	48.5	55.2	48.1	57.7
C10	45.0	37.4	41.8	38.5
C11	154.3	154.8	152.8	152.6
C12	167.76	167.70	164.28	164.15
C13	166.07	166.26	165.80	165.81
C14	2.01	1.47	3.17	1.70
C15	160.70	160.31	160.73	160.66
C16	164.62	167.56	165.59	167.78
C17	133.33	133.02	133.12	133.31
C10a	103.03	102.15	104.89	101.42
C 4a	124.97	118.63	121.08	122.46
C 4b	134.57	138.30	135.87	137.72
C 8a	147.83	147.71	149.01	148.13

S3.3. DP4+ results of compound 2.

Table S3. DP4+ results obtained using experimental data of compound 2 versus isomers 2a, 2b, 2c, and 2d.

Functional	Solvent?	Basis Set	Type of Data			
mPW1PW91	PCM	6-311+G(d, p)	Shielding Tensors			
	Isomer 1	Isomer 2	Isomer 3	Isomer 4	Isomer 5	Isomer 6
sDP4+ (H data)	97.80%	0.49%	0.84%	0.88%	-	-
sDP4+ (C data)	100.00%	0.00%	0.00%	0.00%	-	-
sDP4+ (all data)	100.00%	0.00%	0.00%	0.00%	-	-
uDP4+ (H data)	99.97%	0.03%	0.00%	0.00%	-	-
uDP4+ (C data)	100.00%	0.00%	0.00%	0.00%	-	-
uDP4+ (all data)	100.00%	0.00%	0.00%	0.00%	-	-
DP4+ (H data)	100.00%	0.00%	0.00%	0.00%	-	-
DP4+ (C data)	100.00%	0.00%	0.00%	0.00%	-	-
DP4+ (all data)	100.00%	0.00%	0.00%	0.00%	-	-
